# TBC1D22B Regulates ER‐to‐Golgi Trafficking via RAB1B Inactivation and Promotes Oncogenic Programs in Breast Cancer

**DOI:** 10.1002/advs.202502269

**Published:** 2025-08-29

**Authors:** Flavia Martino, Mariadomenica Lupi, Alessandra Murabito, Fabio Bedin, Giulia Villari, Linda Andreoli, Stefano Freddi, Bronislava Matoskova, Rosa Pennisi, Stella Fontana, Amir Fardin, Gaelle Boncompain, Franck Perez, Federico Bussolino, Alessandro Cuomo, Sara Sigismund, Letizia Lanzetti

**Affiliations:** ^1^ Department of Oncology University of Torino Medical School Candiolo Torino 10060 Italy; ^2^ Candiolo Cancer Institute FPO – IRCCS Candiolo 10060 Torino Italy; ^3^ San Raffaele Telethon Institute for Gene Therapy (SR‐Tiget) IRCCS San Raffaele Scientific Institute Milan 20132 Italy; ^4^ IEO European Institute of Oncology IRCCS Milan 20139 Italy; ^5^ Department of Oncology and Hematology‐Oncology University of Milan Milan 20142 Italy; ^6^ Institut Curie PSL Research University Sorbonne Université Centre National de la Recherche Scientifique CNRS UMR144 Paris 75005 France; ^7^ Institut Neuromyogene CNRS/UCBL UMR5261 INSERM U1315 Université Claude Bernard Lyon1 Lyon France

**Keywords:** breast cancer, ER‐to‐Golgi transport, RAB1B, RabGAP, RUSH, TBC1D22B

## Abstract

TBC1D22B is a GTPase‐activating protein (GAP) associated with poor prognosis in breast cancer (BC). Using complementary proximity‐labeling and co‐immunoprecipitation proteomics, the TBC1D22B interactome in BC cells is defined, revealing strong enrichment in components of the ER‐to‐Golgi trafficking machinery, endosomal transport, and adhesion‐related pathways. Functional assays, using the Retention Using Selective Hooks (RUSH) system, demonstrate that TBC1D22B inhibits ER‐to‐Golgi transport in a GAP‐dependent manner. Mechanistic studies identify RAB1B as a direct target of TBC1D22B, and RAB1B silencing phenocopies the trafficking defects caused by TBC1D22B overexpression. In 3D culture, TBC1D22B promotes spheroid growth in a manner dependent on its GAP activity and not replicated by its paralog TBC1D22A. Transcriptomic profiling reveals that TBC1D22B overexpression triggers repression of a core module of extracellular matrix and adhesion‐related genes, consistent with altered secretory activity. Importantly, this transcriptional program is also evident in primary Luminal BC with high TBC1D22B expression, highlighting a conserved and functionally relevant signature. Together, these findings establish TBC1D22B as a regulator of ER‐to‐Golgi trafficking via RAB1B and implicate it in oncogenic transcriptional remodeling and tumor growth.

## Graphical abstract

TBC1D22B, a Golgi‐localized RabGAP linked to poor prognosis in breast cancer, inhibits ER‐to‐Golgi transport via RAB1B inactivation. This disrupts secretion, drives oncogenic transcriptional reprogramming, and promotes tumor growth. These findings indicate that TBC1D22B connects membrane trafficking to transcriptional control and cancer progression.

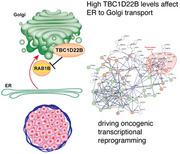

## 1 Introduction

The biogenesis and transport of vesicles require GTPases belonging to the RAB family, which, in mammalian cells, includes more than 60 members.^[^ ^1^ ^]^ RABs act as molecular switches, cycling between GTP‐bound active and GDP‐bound inactive states. The switch to the inactive state occurs when a GTPase‐activating protein (RabGAP) binds to the RAB and stimulates GTP hydrolysis.^[^ ^2^ ^]^ The catalytic activity of RabGAPs resides in a conserved domain, called the TBC domain, which is present in 45 proteins in humans.^[^ ^2^ ^]^ TBC domains accelerate the slow intrinsic GTP hydrolysis rate of RABs by providing two catalytic residues *in trans*: an arginine‐based finger and a glutamine finger. Mutations of these residues result in a strong impairment of the RabGAP catalytic activity.^[^ ^3^ ^]^ The catalytic properties of RabGAPs are rather promiscuous: many RabGAPs function on more than one RAB,^[^ ^4^ ^]^ and many RABs can be regulated by multiple RabGAPs.^[^ ^4^, ^5^ ^]^

In addition to their enzymatic/regulatory function, RabGAPs can act as RAB effectors.^[^ ^6^ ^]^ This dual role is exemplified by RUTBC2, which stimulates GTP hydrolysis on RAB36 while also binding to the active form of RAB9A without affecting its enzymatic activity – a key characteristic of RAB effectors.^[^ ^7^ ^]^ Thus, RabGAPs can act both as negative and positive regulators of RAB activity. An additional layer of complexity arises from the fact that approximately one‐third of TBC domain‐containing proteins are enzymatically impaired due to mutations in the critical arginine and/or glutamine catalytic residues. However, these proteins might still retain the ability to bind their cognate GTPases and function as effectors rather than regulators.^[^ ^2^, ^7^, ^8^ ^]^

By regulating membrane trafficking, RabGAP proteins contribute to many cellular processes that are commonly subverted in pathological conditions, most notably cancer.^[^ ^9^ ^]^ We previously conducted a comprehensive analysis of RabGAP involvement in breast cancer (BC), identifying several whose expression levels correlate with aggressive disease and independently predict prognosis, regardless of other clinical and molecular prognostic markers.^[^ ^10^ ^]^ In particular, high TBC1D22B expression is an independent predictor of poor prognosis in Luminal BC^[^ ^10^ ^]^ – the most common molecular subtype, for which reliable markers to guide therapy decisions are urgently needed.^[^ ^11^ ^]^

Little is known about TBC1D22B. TBC1D22B, and its paralogue TBC1D22A, localize to the Golgi apparatus and interact with the Golgi‐resident protein Acyl‐CoA binding domain containing 3 (ACBD3).^[^ ^12^ ^]^ ACBD3, whose overexpression correlates with poor prognosis in BC,^[^ ^13^ ^]^ serves as a scaffold for assembling a large multiprotein complex, containing Golgin45, GRASP55 and TBC1D22, at the medial Golgi.^[^ ^12a^ ^]^ Although overexpression of both TBC1D22B and TBC1D22A disrupts the ER‐Golgi Intermediate Compartment (ERGIC),^[^ ^14^ ^]^ only TBC1D22A has been shown to inhibit the anterograde transport of G protein‐coupled receptors (GPCRs) from the ER to the Golgi.^[^ ^15^ ^]^ This suggests that the two TBC1D22 isoforms regulate the transport of distinct cargoes in the early secretory pathway.

Given the impact of TBC1D22B alterations in BC, the present study aims to elucidate its biological role and the underlying molecular mechanisms.

## 2 Results

### 2.1 The TBC1D22B Interactome

To gain insights into the biological role of TBC1D22B, we characterized its interactome using a dual approach based on proximity‐labeling, which captures in vivo interactors, and co‐immunoprecipitation (co‐IP), which identifies physical binding partners.

For proximity‐labeling proteomics, lentiviral‐based constructs were engineered to express the ascorbic acid peroxidase, APEX2, fused to TBC1D22B or to a GAP‐inactive mutant in which the catalytic residues (arginine 274 and glutamine 309) were mutated to alanine (henceforth RQ) (Figure S1A, Supporting Information, left). The constructs were transduced into BT549 cells, generating stable cell populations expressing the two fusion proteins at comparable levels (Figure S1A, Supporting Information, right). An empty vector (EV) control was also included (Figure S1A, Supporting Information, right). Consistent with the Golgi localization of endogenous TBC1D22B (Figure S1B, Supporting Information), APEX2‐TBC1D22B and ‐RQ exhibited biotinylating activity predominantly at the Golgi (Figure S1C, Supporting Information). Covalent labeling of proteins in close proximity to TBC1D22B was achieved using a modified version of the protocol described by Hung et al.,^[^ ^16^ ^]^ incorporating additional negative controls in which parallel cultures were treated with H_2_O_2_ alone (Figure S1D, Supporting Information). Following denaturing cell lysis, biotinylated proteins were recovered using streptavidin‐coated magnetic beads (Figure S1D, Supporting Information) and analyzed by mass spectrometry.

Proteins of interest were identified following the scheme in Figure S1E (Supporting Information) (complete proteomes are listed in Table S1 (Supporting Information), with extended data and detailed analyses in Table S2, Supporting Information). From TBC1D22B‐expressing cells, we identified 93 significantly enriched proteins (Table S2, Supporting Information, sheets 1–3 and Experimental section), and 333 unique proteins, i.e., proteins not detected in any negative controls (Table S2, Supporting Information, sheets 4–6). From RQ‐expressing cells, we identified 107 significantly enriched proteins (Table S2, Supporting Information, sheets 7–9) and 330 unique proteins (Table S2, Supporting Information, sheets 10–12).

Next, a “crapome filter” was applied^[^ ^17^ ^]^ to exclude proteins frequently detected as non‐specific contaminants in affinity purification mass spectrometry experiments [http://www.crapome.org/]. The “significantly enriched proteins” were heavily contaminated: 75/93 proteins (80.6%) in the TBC1D22B group and 84/107 proteins (78.5%) in the RQ group were classified as crapome (Table S2, Supporting Information, sheet 13). This observation suggests that, under highly denaturing conditions, most “enriched” proteins were contaminants. After excluding these proteins, 18 TBC1D22B‐ and 23 RQ‐proteins remained (Table S2, Supporting Information, sheet 13). However, it is possible that some true interactors might have been discarded in this filtering step. For example, we showed that EGFR, which was significantly enriched in the TBC1D22B group and “unique” in the RQ group, interacted with TBC1D22B and RQ in co‐immunoprecipitation experiments, albeit at low stoichiometry (Figure S1F, Supporting Information). However, given our goal of identifying biological pathways and cellular processes involving TBC1D22B, we prioritized stringently selected “unique” hits (Figure S1E, Supporting Information).

**Figure 1:**
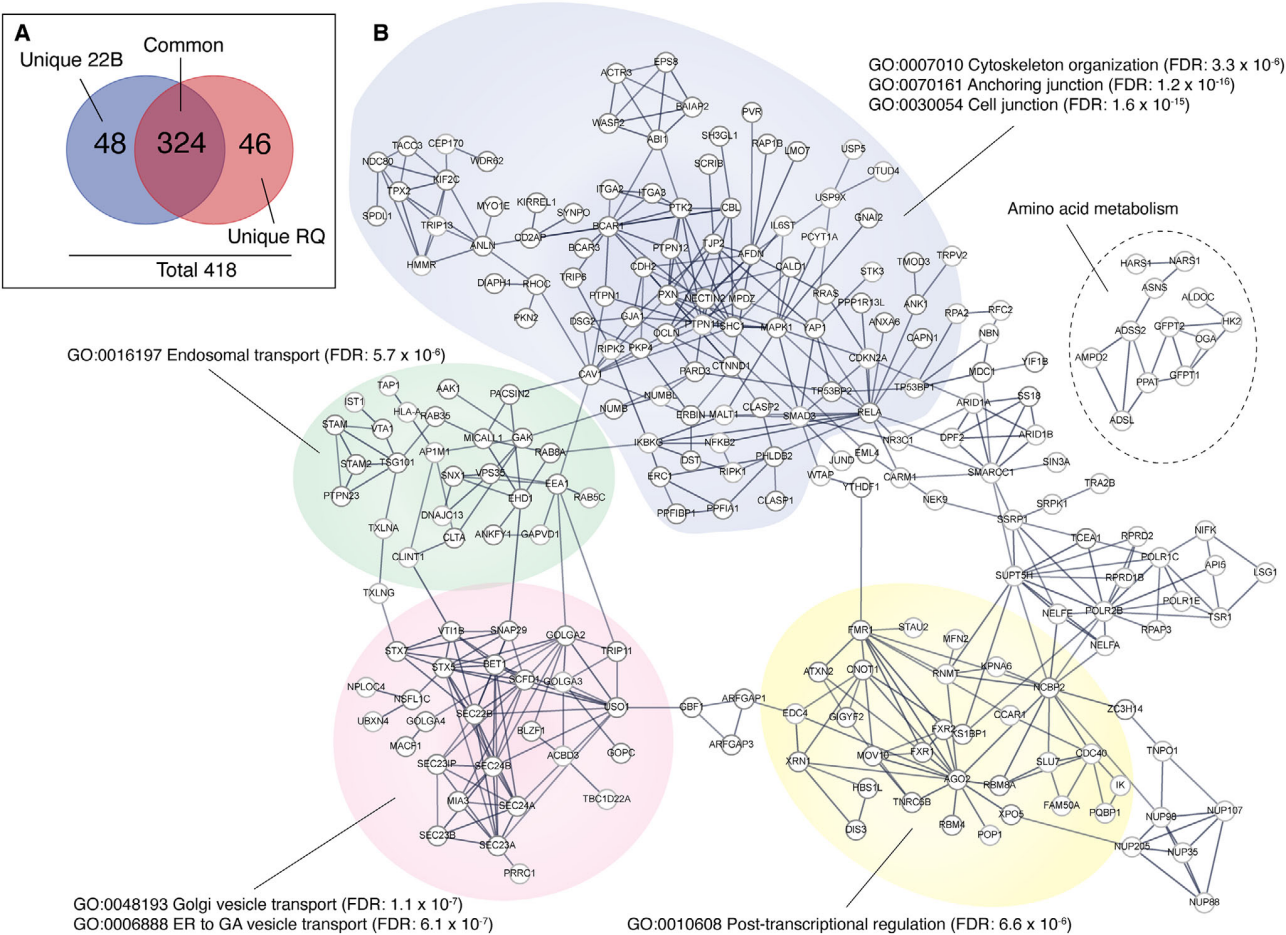
Pathway analysis of the TBC1D22B proximity proteome. A) Venn diagram showing the overlap between proteins detected in the proximity proteomes of TBC1D22B (22B) or of its RQ mutant. B) Pathway analysis of the proximity proteome. The analysis was performed with STRING (https://string‐db.org/),^[^ ^18^ ^]^ with the following settings: active interaction sources, textmining, experiments, databases, co‐expression; minimum required interaction score, high confidence (0.7). Disconnected nodes and smaller networks were omitted. EGFR was also removed, as it was a strong attractor that caused all sub‐networks to merge into a single network, making it difficult to visualize. Relevant subnetworks are highlighted (with their FDRs in parentheses) as obtained from the “analysis” function of STRING; the complete list of GO terms is provided in Table S2 (Supporting Information), sheet 15, obtained by Enrich (https://maayanlab.cloud/Enrichr/). The PPI (protein:protein interaction) enrichment p‐value of the network is <10^−16^.

The crapome analysis of “unique” proteins revealed a lower level of contamination: only 60/333 (18.0%) proteins in the TBC1D22B group, and 83/330 (25.1%) proteins in RQ group (Table S2, Supporting Information, sheet 13). The remaining TBC1D22B‐ and‐RQ unique proteins (273 and 247, respectively, Table S2, Supporting Information, sheet 13) were retained for further analysis (Figure S1E, Supporting Information). The overlap between the TBC1D22B and RQ proteomes was limited to 102 proteins (Table S2, Supporting Information, sheet 14‐stringent overlap). However, our strict criteria, requiring proteins to be present in all four TBC1D22B and RQ replicates and absent from all controls (4 EV replicates and 8 H_2_O_2_‐only replicates), might have underestimated common hits. Relaxing the stringency slightly, allowing proteins to appear in one replicate of one control, increased the overlap to 324/418 (77.5%) non‐redundant proteins (**Figure** **1**A; Table S2, Supporting Information, sheet 14‐less stringent overlap). Notably, the only validated interactor of TBC1D22B, i.e., ACBD3,^[^ ^12^ ^]^ was among the common proteins and was shown to physically interact with both TBC1D22B and RQ (Figure S1G, Supporting Information).

We concluded that the proximity interactomes of 22B and RQ are highly similar. We suspect that most of the TBC1D22B‐only or RQ‐only proteins (48 and 46, respectively, Figure 1A; Table S2, Supporting Information, sheet 14) might be “common” proteins, since they would fall into this category if two mismatched controls were allowed (data not shown). Therefore, we used the entire set of 418 proteins for subsequent pathway analysis.

Pathway analysis was performed using STRING, which visualizes networks of protein interactions, as well as assigning ontologies to the identified proteins.^[^ ^18^ ^]^ A dense network of interactions was identified (Figure 1B). Enrichment of specific ontologies was evident, with highly significant results, particularly in the categories of ER‐to‐Golgi transport and Golgi vesicle transport, consistent with the predominant subcellular localization of TBC1D22B (Figure S1B, Supporting Information). Additionally, proteins involved in endosomal transport were also enriched (Figure 1B). Another prominent sub‐network included proteins associated with cytoskeleton organization, cell‐cell junctions, and cell‐matrix interactions (Figure 1B; the complete list of ontology terms is provided in Table S2, Supporting Information, sheet 15).

The proximity‐labeling approach cannot distinguish between proteins that are labeled due to direct physical interaction with the bait or those that localize near the bait within the same subcellular domain. To address this issue, mass‐spectrometry was performed on proteins co‐immunoprecipitated from BT549 cells expressing HA‐tagged‐TBC1D22B with an anti‐HA antibody. EV‐transduced cells were used as controls, and mild lysis and binding conditions were applied (complete proteomes are listed in Table S3, Supporting Information, with extended data and detailed analyses in Table S4, Supporting Information).

**Figure 2:**
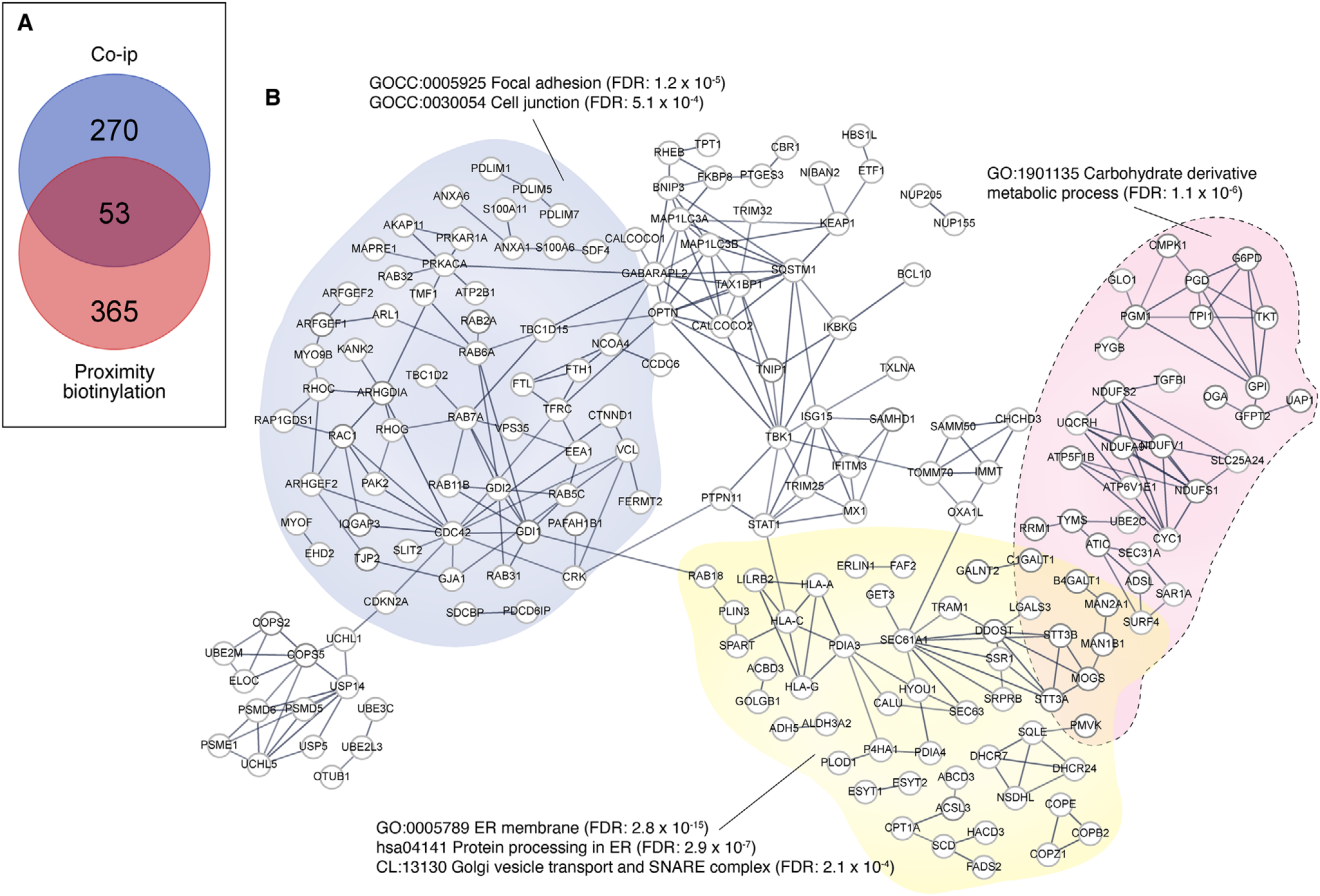
Pathway analysis of the TBC1D22B physical interactome. A) Venn diagram showing the overlap between the TBC1D22B proximity proteome and the physical (co‐ip) proteome. The overlap between the two proteomes was highly significant, p = 7.6×10^−7^ in a hypergeometric distribution assuming a conservative proteome of 5000 detectable species, performed online [https://systems.crump.ucla.edu/hypergeometric]. B) Pathway analysis of the TBC1D22B physical proteome. The analysis was performed using STRING (https://string‐db.org/),^[^ ^18^ ^]^ with the following settings: active interaction sources, textmining, experiments, databases, co‐expression; minimum required interaction score, high confidence (0.7). Disconnected nodes and smaller networks were omitted. Relevant subnetworks are highlighted as obtained from the “analysis” function of STRING (with their FDRs in parentheses); the complete list of GO terms is provided in Table S4 (Supporting Information), sheet 4, obtained by Enrich (https://maayanlab.cloud/Enrichr/). The PPI enrichment p‐value of the network is <10^−16^.

Using the same stepwise filtering strategy applied to proximity‐labeling experiments, 361 significantly enriched and 108 unique proteins were identified in TBC1D22B co‐immunoprecipitates (Table S4, Supporting Information, sheets 1 and 2). The abundance of the significantly enriched proteins is likely due to the mild non‐denaturing conditions used in co‐IP experiments to preserve protein‐protein interactions. Crapome contamination was substantially lower in these experiments compared to proximity‐labeling experiments. In both the significantly enriched and the unique lists, 38.8% and 5.6% of hits, respectively, were excluded after applying the crapome filter (Table S4, Supporting Information, sheet 3). Overall, 53/323 (15.8%) proteins co‐immunoprecipitating with TBC1D22B were also detected in the proximity interactome (p = 7.6 × 10^−7^) (**Figure** **2**A; Table S4, Supporting Information, sheet 3). These data indicate that a significant portion of the two interactomes overlap.

Since the significantly enriched and unique co‐immunoprecipitating lists showed a comparable degree of overlap with the proximity interactome, we reasoned that they were likely not differently biased by potential contaminants. Therefore, we used the combined co‐IP protein list (*n* = 323) for pathway analysis (Figure 2B). Consistent with the pathway analysis of the proximity interactome, terms related to ER and Golgi dynamics and transport, as well as cell‐cell and cell‐matrix interactions, were highly significant in the physical (co‐IP) interactome (Figure 2B, the complete list of ontology terms is provided in Table S4, Supporting Information, sheet 4).

### 2.2 TBC1D22B Inhibits ER‐to‐Golgi Transport

The TBC1D22B proximity and physical interactomes show significant enrichment in proteins involved in the ER‐to‐Golgi transport and secretory pathways (Figures 1 and 2), suggesting a role for TBC1D22B in regulating the anterograde secretory pathway. To obtain functional evidence, we utilized the Retention Using Selective Hooks (RUSH) system.^[^ ^19^ ^]^ The RUSH system allows molecules moving from donor to acceptor compartments to be tracked by co‐expressing two proteins:

i) A fluorescently tagged reporter, consisting of a cargo protein, fused to a streptavidin‐binding peptide (SBP), which traffics from a donor to an acceptor compartment. We used GFP‐tagged glycosylphosphatidylinositol (SBP‐GFP‐GPI, here indicated as GFP‐GPI) as a cargo reporter.
ii) A “hook” molecule, stably anchored in the donor compartment and fused to a core streptavidin. The hook retains the fluorescent cargo in the donor compartment through the interaction between streptavidin and the SBP moiety of the reporter cargo. The addition of biotin disrupts this interaction, allowing the reporter to detach from the hook and synchronously travel to the acceptor compartment.^[^ ^19^ ^]^ We used a KDEL sequence that specifically localizes to the ER as the hook (Str‐KDEL).

We first investigated the effects of TBC1D22B overexpression on the anterograde pathway by live cell imaging transiently co‐transfecting the RUSH construct (Str‐KDEL_SBP‐GFP‐GPI) with mCherry‐tagged TBC1D22B, or RQ, in BT549 cells. Control cells were transfected with the RUSH construct alone (CTR) (Movies S1–S3 and Figure S2, Supporting Information). In the absence of biotin, the RUSH reporter localized to diffuse reticular structures throughout the cytoplasm, consistent with ER localization (Movies S1–S3, Supporting Information). Upon the addition of biotin, the reporter relocalized to the Golgi in ≈10–25 min in CTR cells, followed by its disappearance from the Golgi and trafficking to the cell periphery (Movie S1, Supporting Information‐CTR), as previously reported.^[^ ^19^ ^]^ In mCherry‐TBC1D22B cells, relocalization of the reporter to the Golgi was delayed, becoming detectable after 50 min (Movie S2, Supporting Information‐TBC1D22B). Expression of the mCherry‐RQ mutant did not affect ER‐to‐Golgi transport, with kinetics comparable to CTR cells (≈25 min, Movie S3, Supporting Information‐RQ). Post‐Golgi trafficking of the reporter was delayed both in TBC1D22B and RQ cells (Movie S2, Supporting Information‐TBC1D22B and Movie S3, Supporting Information‐RQ).

**Figure 3:**
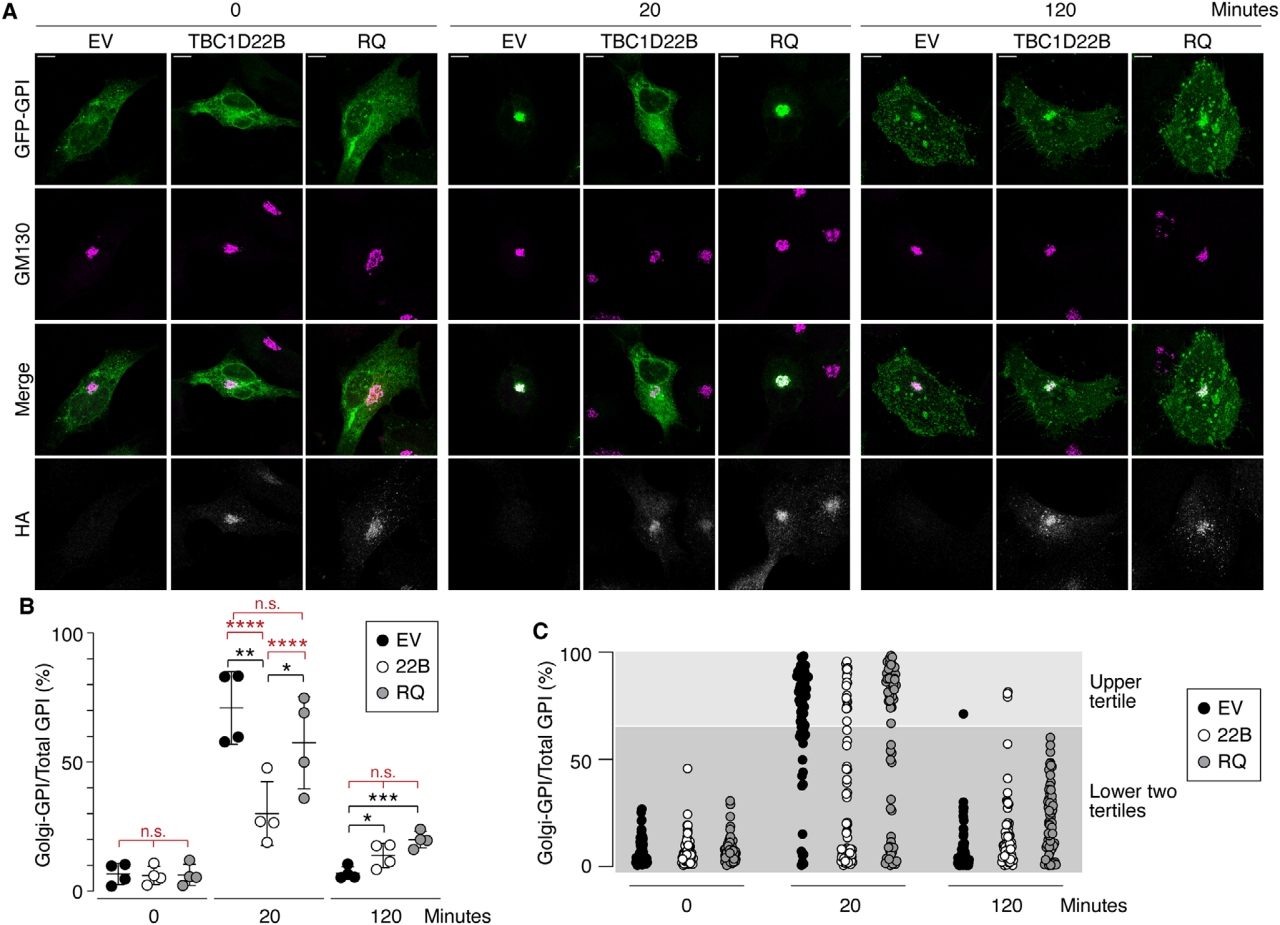
Effect of TBC1D22B overexpression on ER‐to‐Golgi transport. A) RUSH assays in BT549 cells stably expressing HA‐TBC1D22B or HA‐TBC1D22B‐RQ (RQ) or empty vector (EV) control. Cells were transfected with the Str‐KDEL_SBP‐EGFP‐GPI RUSH construct, then treated with biotin to release the GFP‐GPI reporter. Time points after biotin addition are indicated on top. Images show GFP‐GPI epifluorescence (green), and IF staining for the Golgi marker GM130 (magenta) and TBC1D22B (anti‐HA staining, grey). Merge results from the overlap of the GFP‐GPI epifluorescence and the GM130 staining. Bar, 10 µm B) Quantitation of the experiment shown in A. The percentage of Golgi‐localized GFP‐GPI, normalized to the total GFP‐GPI signal per cell, is plotted at time 0, 20 and 120 min after biotin addition. Data represent the mean ± SD of four independent experiments, in which ≈15–20 cells/experiment/condition were analyzed. Significance (black asterisks) was calculated with the t‐test. Red asterisks refer to the statistical analysis performed on the tertile distribution shown in panel C. *, p < 0.05, **, p < 0.01, ***, p < 0.001, **** p < 0.0001, n.s., not significant. C) Stratification of the entire dataset of % Golgi‐GPI/Total‐GPI per cell is shown (at least 64 cells/condition). The upper tertile shows cells with a Golgi‐GPI > 66%; the lower two tertiles show cells with a Golgi‐GPI ≤ 66%. Statistical analysis was performed with the Fisher's test and is reported in Table S5 (Supporting Information) (also as red asterisks in panel B).

Next, we quantified these findings on fixed BT549 cells expressing HA‐tagged TBC1D22B, or RQ, or the empty vector (EV) by measuring the amount of GFP‐GPI co‐localizing with the Golgi marker GM130, normalized to the total GFP‐GPI signal per cell (**Figure** **3**A–C). In the absence of biotin, GFP‐GPI showed negligible co‐localization with GM130 in EV control cells and HA‐TBC1D22B or HA‐RQ expressing cells (Figure 3A,B). Twenty min after biotin addition, GFP‐GPI predominantly localized to the Golgi in EV and HA‐RQ cells (Figure 3A,B). However, in HA‐TBC1D22B cells, GFP‐GPI accumulation at the Golgi was substantially reduced (Figure 3A,B). Co‐localization of GFP‐GPI with GM130 decreased 120 min after biotin treatment, reflecting the post‐Golgi trafficking of the cargo (Figure 3A,B). Comparing the average GFP‐GPI signal at the Golgi across conditions, statistical significance was observed between EV and TBC1D22B at 20 min (indicated by black asterisks in Figure 3B). Smaller but significant differences were also detected between EV and either TBC1D22B or RQ at 120 min. When analyzing all the measurements in a stratified distribution, based on tertiles of intensity of the GFP‐GPI signal at the Golgi, (Figure 3C, upper tertile shows cells with a Golgi‐GPI > 66%; the lower two tertiles show cells with a Golgi‐GPI ≤ 66%), statistical significance was achieved for EV versus TBC1D22B and TBC1D22B versus RQ comparisons only at 20 min (red asterisks in Figure 3B; Table S5, Supporting Information). Similar results were obtained by performing the RUSH experiments in a different BC cell line, the CAL120 cells (Figure S3 and Table S6, Supporting Information). These data indicate that overexpression of TBC1D22B delays ER to Golgi transport in a GAP‐dependent manner. Small differences are also found in post‐Golgi trafficking upon overexpression of TBC1D22B and RQ, but they do not retain significance when cells are stratified based on the intensity of the reporter at the Golgi.

**Figure 4:**
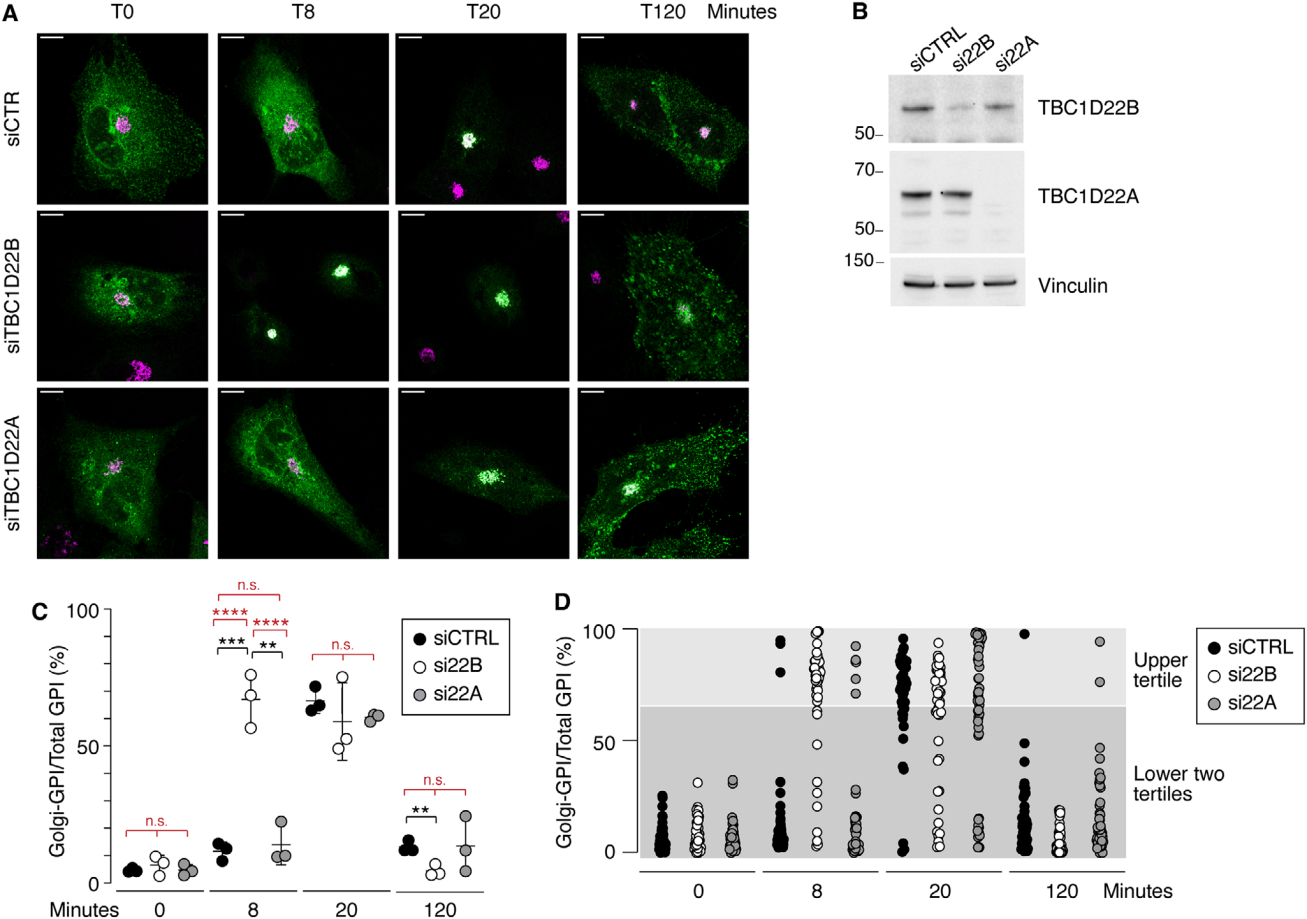
Silencing of TBC1D22B, but not of TBC1D22A, accelerates GFP‐GPI ER‐to‐Golgi transport. A) RUSH assays in BT549 cells silenced as indicated on the left. Cells were transfected with the Str‐KDEL_SBP‐EGFP‐GPI construct, then treated with biotin to release the GFP‐GPI reporter. Time points after biotin addition are indicated on top. Merged images show GFP‐GPI epifluorescence (green) and IF staining for the Golgi marker GM130 (magenta). Bar, 10 µm. B) Effective silencing of TBC1D22B and TBC1D22A was assayed by immunoblotting (IB) of total cellular lysates from the samples treated as in A (indicated on top) with the antibodies shown on the right (vinculin, loading control). C) Quantitation of the experiment shown in A. The percentage of Golgi‐localized GFP‐GPI, normalized to the total GFP‐GPI signal per cell, is plotted at time 0, 8, 20 and 120 min after biotin addition. Data represent the mean ± SD of three independent experiments, in which ≈10–16 cells/experiment/condition were analyzed. Significance (black asterisks) was calculated with the t‐test. Red asterisks refer to the statistical analysis performed on the tertile distribution shown in panel D. **, p < 0.01, ***, p < 0.001, ****, p < 0.0001, n.s., not significant. D) Tertile analysis. The upper tertile shows cells with a Golgi‐GPI > 66%; the lower two tertiles show cells with a Golgi‐GPI ≤ 66%. Statistical analysis was performed with the Fisher's test and is reported in Table S7 (Supporting Information) (also as red asterisks in panel C).

Finally, to explore the physiological involvement of TBC1D22B in ER‐to‐Golgi transport we analyzed the effects of transient silencing (detailed in Experimental section) of endogenous TBC1D22B on GFP‐GPI transport. As a specificity control, we silenced TBC1D22A, the closest TBC1D22B paralogue (see Introduction). Silencing of TBC1D22B (siTBC1D22B), but not of TBC1D22A (siTBC1D22A), accelerated ER‐to‐Golgi trafficking of the GFP‐GPI reporter (**Figure** **4**A–C). Faster kinetics of post‐Golgi trafficking were also observed at 120 min in the TBC1D22B silenced cells likely owing to overall accelerated traffic of the reporter (Figure 4A–C). Tertile analysis confirmed accelerated ER‐to‐Golgi transport of GFP‐GPI upon depletion of TBC1D22B (Figure 4D; Table S7, Supporting Information, and red asterisks in Figure 4C).

We concluded that TBC1D22B is a physiological inhibitor of ER‐to‐Golgi transport.

### 2.3 TBC1D22B Regulates ER‐to‐Golgi Transport Through its Modulation of RAB1B Activity

Our findings indicate that the GAP activity of TBC1D22B suppresses ER‐to‐Golgi transport, suggesting that TBC1D22B inhibits trafficking between these two compartments by switching off one or more RAB GTPases. If so, the silencing of RAB substrate(s) should phenocopy the overexpression of TBC1D22B.

**Figure 5:**
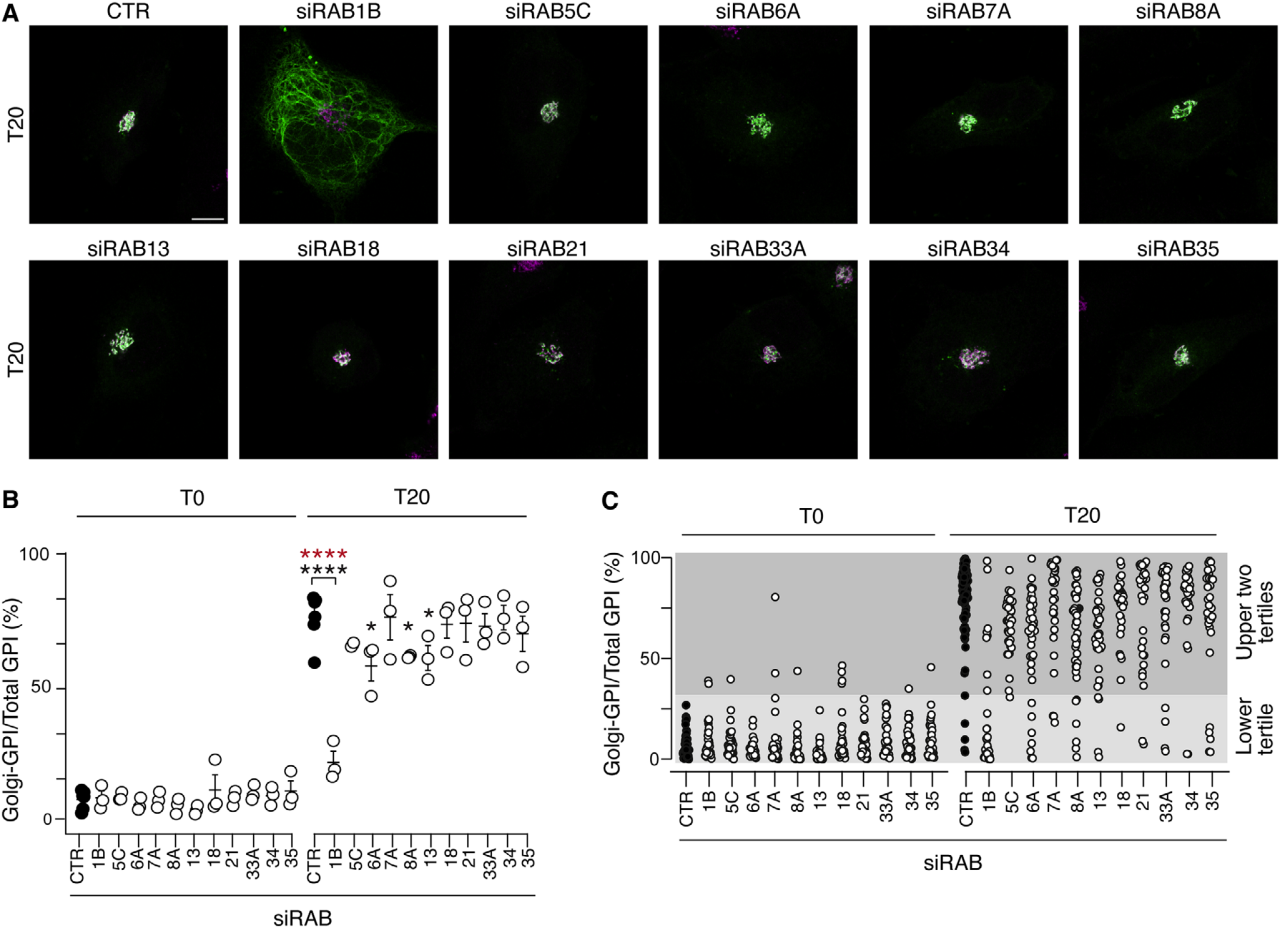
Effect of RAB silencing on ER‐to‐Golgi transport. A) RUSH assays in BT549 cells silenced for the indicated RABs. Cells were transfected with the Str‐KDEL_SBP‐EGFP‐GPI construct, then treated with biotin to release the GFP‐GPI reporter. Merged images show GFP‐GPI epifluorescence (green) and IF staining for the Golgi marker GM130 (magenta) at the 20‐min time point (T20) after biotin addition. Bar, 10 µm. Images at T0 are shown in Figure S4 (Supporting Information). B) Quantitation of the experiment shown in B. The percentage of Golgi‐GPI/total‐GPI per cell is plotted at time 0 and 20 min after biotin addition. Data represent the mean ± SD of three independent experiments, in which ≈10‐12 cells/experiment/condition were analyzed. Significance (black asterisks) was calculated with the t‐test. The red asterisks refer to the statistical analysis performed on the tertile distribution shown in panel C. *, p < 0.05, ****, p < 0.0001. C) Tertile analysis. The lower tertile shows cells with a Golgi‐GPI < 33%; the upper two tertiles show cells with a Golgi‐GPI > 33%. Statistical analysis was performed with the Fisher's test and is reported in Table S9 (Supporting Information) (also as red asterisks in panel B).

To identify potential RAB targets of TBC1D22B, we inspected the proximity and physical interactomes of the protein. Nineteen and 10 RABs were present in the proximity and physical interactomes, respectively, with 7 overlapping ones. We used the proximity interactome list as the starting point for the selection, because of its higher granularity (interactomes obtained both with TBC1D22B and RQ) and redundance of controls (8 negative controls). Through a quantitative scoring matrix (see Table S8, Supporting Information), we identified 11 candidates (4 of which were also contained in the physical interactome). The effects of silencing these candidates (Figure S4A, Supporting Information) on ER‐to‐Golgi transport were analyzed in BT549 cells using the RUSH assay. Silencing of RAB1B significantly decreased the relocalization of the GFP‐GPI reporter to the Golgi at the 20‐min time point after biotin addition (**Figure** **5**A–C; Table S9, Supporting Information, time 0 images in Figure S4B, Supporting Information).

Minor delays in ER‐to‐Golgi transport were observed upon RAB6A, RAB8A and RAB13 KD (Figure 5B); however, these delays did not reach significance in the tertile‐based analysis. Thus, RAB1B silencing phenocopies TBC1D22B overexpression. Together with the proximity‐labeling data, these results suggest that TBC1D22B inhibits ER‐to‐Golgi transport through RAB1B inactivation.

**Figure 6:**
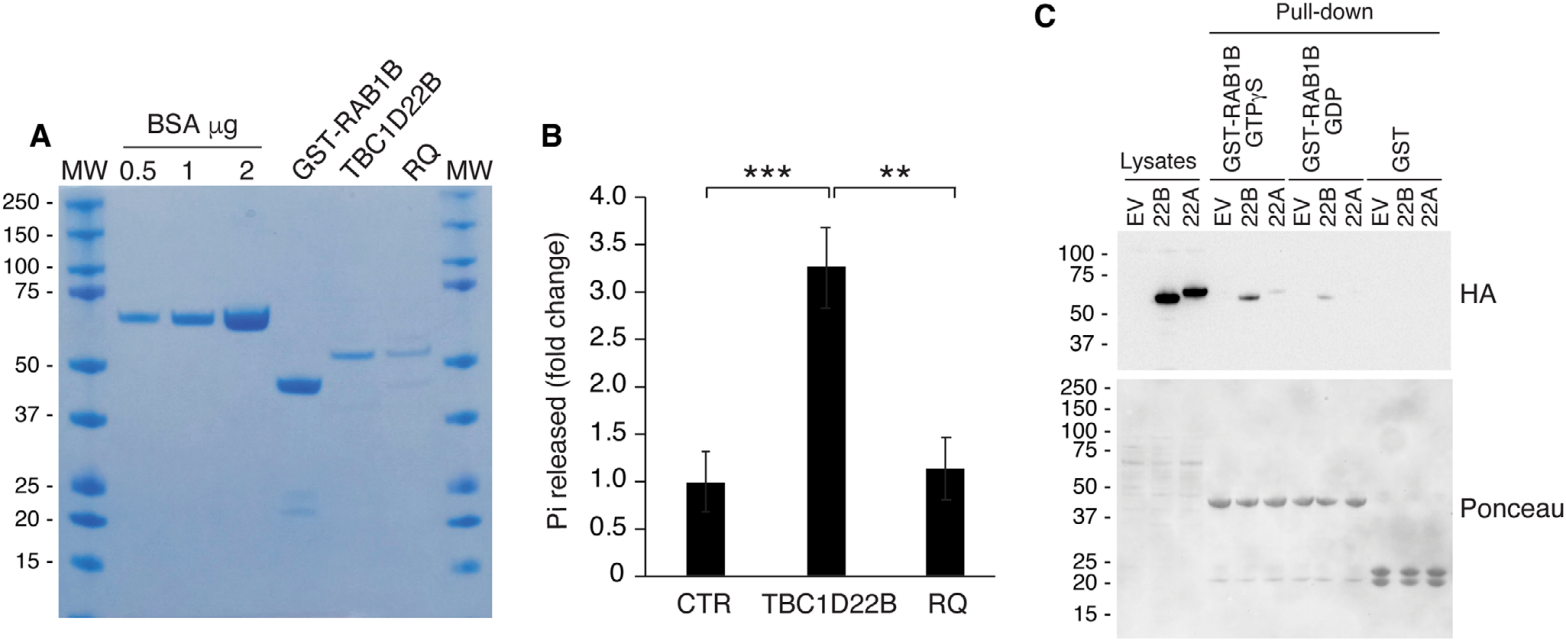
TBC1D22B interacts with RAB1B and stimulates GTP hydrolysis on RAB1B. A) Expression, purification and quantitation of the GST‐RAB1B, TBC1D22B and TBC1D22B‐RQ (RQ) proteins produced in BL21 bacteria. After purification, TBC1D22B and RQ were digested with thrombin to remove the GST moiety. The colloidal blue gel shows aliquots of the purified proteins to evaluate the purity and a standard BSA curve used for quantification. B) GAP assay. The bar graph shows the fold‐change of phosphate (Pi) released by GST‐RAB1B‐GTP in presence of buffer alone (CTR) or TBC1B22B or RQ, normalized to CTR. Bars are the mean ± sem of three independent experiments performed in technical triplicates. Statistical analysis was performed with the t‐test, p values: **, p < 0.01, ***, p < 0.001. C) RAB1B pull‐down assay. Purified GST‐RAB1B loaded with either GTP‐γ‐S or GDP, or GST alone were incubated with total cellular lysates from BT549 cells expressing the empty vector (EV), HA‐TBC1D22B or HA‐TBC1D22A as described in the Experimental section. Input lanes are 5 µg of total cellular lysates from the indicated cells populations. The upper panel is the western blot of the pull‐down assay immunoblotted with the anti‐HA antibody; the lower panel is the Ponceau staining of the same membrane.

To test whether TBC1D22B inactivates RAB1B, we measured the ability of TBC1D22B to stimulate GTP hydrolysis on this GTPase in in vitro GAP assays. We purified recombinant, bacterially‐expressed TBC1D22B and RQ and assessed their enzymatic GAP activity on purified RAB1B (**Figure** **6**A,B). TBC1D22B displayed significant GAP activity toward RAB1B, while the RQ mutant did not (Figure 6B).

RabGAP‐mediated catalysis involves the physical interaction of the RabGAP with the GTP‐loaded target RAB.^[^ ^2^ ^]^ We tested this interaction in in vitro pull‐down assays. The GST‐RAB1B fusion protein was loaded with either GTP‐γ‐S (a non‐hydrolysable version of GTP that locks RAB1B in its GTP active form) or GDP and used as a bait to capture proteins from cellular lysates expressing either HA‐TBC1D22B or HA‐TBC1D22A. RAB1B was able to interact with TBC1D22B significantly better in the GTP‐γ‐S loaded form than in the GDP‐bound form, while negligible binding was detected with HA‐TBC1D22A (Figure 6C).

Together, these findings confirm that TBC1D22B functions as a GAP for RAB1B, supporting the role of a TBC1D22B‐RAB1B‐dependent mechanism in the regulation of ER‐to‐Golgi trafficking.

### 2.4 Relevance of TBC1D22B to Breast Carcinogenesis

The overarching goal of this study was to delineate the molecular circuitry regulated by TBC1D22B, given its previously demonstrated association with breast cancer (BC) progression – specifically, our earlier findings identified high TBC1D22B expression as an independent predictor of poor prognosis in Luminal BC.^[^ ^10^ ^]^ To investigate the relevance of the high expression of TBC1D22B in breast carcinogenesis, we employed a dual approach combining high‐resolution in vitro experimentation with high‐throughput bioinformatic analyses of large BC datasets.

**Figure 7:**
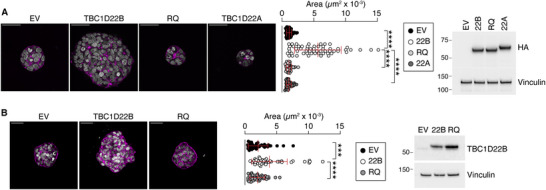
Overexpression of TBC1D22B, but not of TBC1D22A, induces tumor spheroids overgrowth, in a GAP‐dependent manner. BT549 cells stably expressing the empty vector (EV), HA‐tagged TBC1D22B, RQ or TBC1D22A were grown in matrigel for 7 days to form tumor cell spheroids. Spheroids were stained with Phalloidin and DAPI shown respectively in magenta and grey in the merged representative images (left). Images are confocal Z‐stack projections (8 optical sections/image) of spheroids acquired with a 40x objective, zoom 1.5x. Bar, 50 µm. Quantification of the spheroids area, shown in the middle panel, is from 3 independent experiments in which 45–71 spheroids/condition were measured. Mean ± SD are also shown in the plots. Statistical analysis was performed with the t‐test, **** p < 0.0001. The Western blots on the right were IB with the indicated antibodies (vinculin, loading control) and show the expression levels of TBC1D22B, RQ and TBC1D22A. B) CAL120 cells stably expressing the empty vector (EV), the HA‐tagged TBC1D22B, or RQ were grown and stained as in A. Images are confocal Z‐stack projections (8 optical sections/image) of representative spheroids acquired with a 20x objective, zoom 2. Bar, 50 µm. In the middle panel the area of 45 spheroids/condition from 3 independent experiments is shown. Mean ± SD are also shown in the plots. Statistical analysis was performed with the t‐test, p values: ***, p < 0.001, **** p < 0.0001. The Western blot on the right shows the expression levels of TBC1D22B and RQ.

We first assessed whether overexpression of TBC1D22B in breast epithelial cells – mimicking conditions observed in a subset of BCs^[^ ^10^ ^]^ – confers a proliferative advantage. TBC1D22B overexpression significantly enhanced organoid growth in 3D matrigel culture in both the BT549 and CAL120 cell lines (**Figure** **7**A,B). This effect was dependent on the GAP activity of TBC1D22B, as the RQ mutant failed to elicit a comparable phenotype (Figure 7A,B). Notably, overexpression of the paralogous gene TBC1D22A did not enhance spheroid growth (Figure 7A).

**Figure 8:**
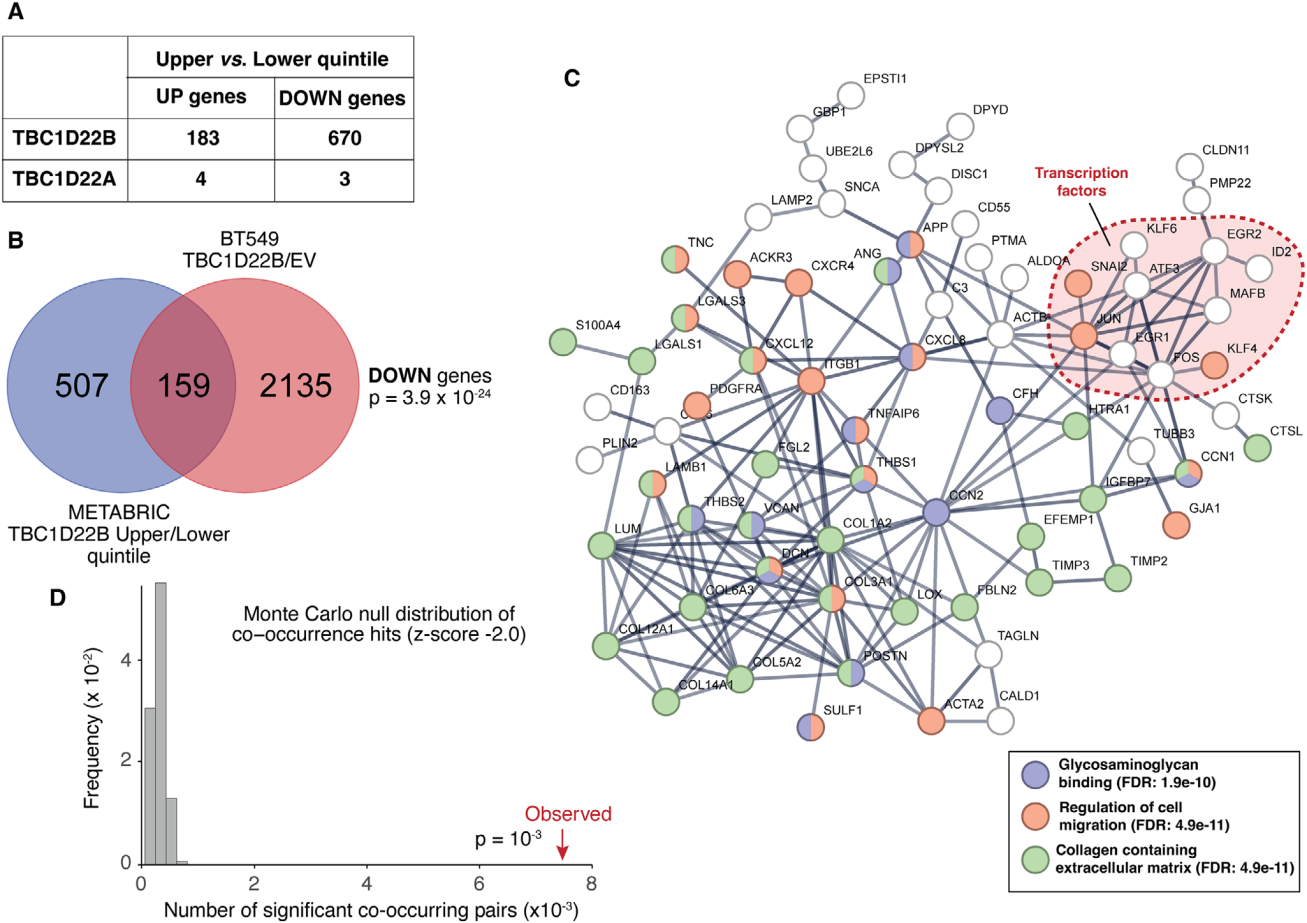
A transcriptional program downregulated by TBC1D22B in BC. A) Number of upregulated (UP) and downregulated (DOWN) genes obtained by comparing the upper versus lower quintile of expression of TBC1D22B and TBC1D22A in the METABRIC dataset. B) Venn diagram showing the overlap of genes downregulated in the comparisons BT549‐TBC1D22B/BT549‐EV and TBC1D22B‐high/TBC1D22B‐low BCs. Only genes present in both datasets were considered (see Table S10, Supporting Information sheet 4, where the list of the common 159 genes is also shown). C) STRING pathway analysis. The analysis was performed with the following settings: active interaction sources, textmining, experiments, databases, co‐expression; minimum required interaction score, high confidence (0.7). Disconnected nodes and smaller networks not connected to the major network were deleted. Some relevant subnetworks are highlighted as obtained from the “analysis” function of STRING; a complete list of GO terms is in Table S10 (Supporting Information) sheet 5, as obtained by Enrich (https://maayanlab.cloud/Enrichr/). The PPI (protein:protein interaction) enrichment p‐value of the network is <10^−16^. D) Montecarlo analysis showing the distribution of co‐occurrence of downregulation (z‐score ‐2.0) of 1000 random sets of 159 genes in the METABRIC dataset versus the 159 genes identified in B (indicated by the red arrow).

To extend these findings to primary tumors, we analyzed transcriptomic data from the METABRIC cohort of BCs. We stratified the Luminal tumors from this cohort (N = 1369) based on TBC1D22B expression levels, since TBC1D22B overexpression predicts prognosis in this subgroup of patients.^[^ ^10^ ^]^ The average expression of TBC1D22B in the upper versus lower quintiles differed by ≈1.9‐fold (q = 3.1 × 10^−^^2^⁴⁷). Differential gene expression analysis (≥1.5‐fold change; q < 0.05) identified 183 upregulated and 670 downregulated genes (Table S10, Supporting Information, Sheet 1; **Figure** **8**A). In contrast, a similar analysis using TBC1D22A, which lacks prognostic value in BC,^[^ ^10^ ^]^ revealed a 1.5‐fold difference in expression (q = 4.1 × 10^−^^2^⁷^3^) between upper and lower quintiles but only 7 differentially expressed genes (4 upregulated, 3 downregulated; Table S10, Supporting Information, Sheet 2; Figure 8A). These findings suggest that TBC1D22B plays a significant role in driving transcriptional changes associated with poor disease prognosis in Luminal BCs, whereas TBC1D22A does not exhibit similar prognostic or transcriptional regulatory effects (Figure 8A).

To further elucidate the molecular consequences of TBC1D22B overexpression, we examined the transcriptome of BT549 cells, in which TBC1D22B promotes 3D growth. Comparison of cells transfected with TBC1D22B versus empty vector (EV) identified 2849 upregulated and 2830 downregulated genes (≥2.0 or <0.5 ‐fold change; q < 0.05; Table S10, Supporting Information, Sheet 3). To ensure compatibility with the METABRIC dataset, we excluded poorly annotated or non‐coding transcripts (e.g., pseudogenes, antisense RNAs, LINCs, LOCs) (Table S10, Supporting Information, Sheet 4). A notable overlap was observed among downregulated genes: 159 genes (23.9%) were significantly repressed in both BT549‐TBC1D22B cells and TBC1D22B‐high Luminal tumors (p = 3.9 × 10^−^^2^⁴; Figure 8B). In contrast, the overlap of upregulated genes was minimal and not statistically significant (*n* = 8, p = 0.074; Table S10, Supporting Information, Sheet 4). These results indicate that TBC1D22B overexpression in vitro recapitulates a significant part of the transcriptional alterations seen in primary tumors and underscore its oncogenic potential.

To identify biological pathways affected by these shared transcriptional changes, we conducted STRING network analysis on the 159 commonly downregulated genes. This revealed a highly interconnected network centered on extracellular matrix organization, glycosaminoglycan binding, and cell motility/adhesion (Figure 8C; Table S10, Supporting Information, Sheet 5). Intriguingly, most of the proteins encoded by the 159 genes are secreted, consistent with TBC1D22B's known role in modulating protein trafficking and secretion. Although the mechanistic link between the effects of TBC1D22B on secretory phenotypes and global transcriptional repression remains unclear, several transcription factors were themselves downregulated in response to TBC1D22B overexpression (Figure 8C), suggesting possible feedback or network effects.

Finally, the identification of 159 commonly downregulated genes in both TBC1D22B‐overexpressing BCs and BT549 cells raised the question of whether these genes are part of a transcriptional program coordinately regulated by TBC1D22B. To address this, we assessed the co‐occurrence of gene downregulation across the METABRIC dataset. Among 12561 gene pairs (from 159 genes), 7354 pairs (58.5%) showed significant co‐occurrence of downregulation (p = 10^−^^3^, Monte Carlo simulation; Figure 8D; Table S10, Supporting Information, Sheet 6). This co‐regulatory signature was independently validated in the TCGA breast cancer dataset, where 7593 pairs (60.4%) displayed similar co‐occurrence patterns (p = 10^−^^3^). These findings support the existence of a TBC1D22B‐regulated transcriptional module, enriched for genes involved in ECM remodeling and adhesion, whose repression may underpin the adverse clinical outcomes observed in TBC1D22B‐overexpressing Luminal BCs.

## 3 Discussion

Here, by integrating a variety of high‐throughput and high‐resolution assays, we demonstrate that TBC1D22B is a Golgi‐resident RabGAP that regulates the secretory pathway by acting as a GAP for RAB1B, and that the overexpression of TBC1D22B, which correlates with aggressive disease course,^[^ ^10^ ^]^ is mechanistically relevant to breast carcinogenesis.

### 3.1 Golgi‐Localized TBC1D22B Links Secretory Pathway Regulation to Cancer‐Relevant Networks

Our dual‐proteomics strategy uncovered a robust network of TBC1D22B‐associated proteins, using both proximity‐labeling and co‐immunoprecipitation approaches. The resulting interactomes showed significant enrichment in protein networks associated with ER‐to‐Golgi transport, endosomal trafficking, cytoskeleton remodeling, and cell adhesion. This is consistent with the predominant localization of TBC1D22B to the Golgi apparatus, where it likely functions to coordinate vesicle trafficking dynamics and membrane identity.

A particularly prominent protein module in the proximity interactome included components of the COPII vesicle machinery, such as SEC23 and SEC24, which drive anterograde ER export, and SEC22B, a v‐SNARE implicated in vesicle fusion.^[^ ^20^ ^]^ Several golgins, including GOLGA2 (GM130), GOLGA3, and GOLGA4, were also detected.^[^ ^21^ ^]^ These proteins are key tethers of ER‐derived vesicles and effectors of RAB1B^[^ ^22^ ^]^ and RAB33.^[^ ^23^ ^]^ Notably, GOLGA2 directly binds active RAB1B to tether COPII vesicles at the cis‐Golgi,^[^ ^24^ ^]^ while GOLGA3 mediates cargo delivery via ACBD3,^[^ ^25^ ^]^ itself a validated interactor of TBC1D22B. The inclusion of TANGO1/MIA3, a large cargo export factor at ER exit sites (ERES), suggests that TBC1D22B may be particularly relevant to the export of bulky secretory proteins, such as ECM components.^[^ ^26^ ^]^

Another major network identified by proximity labeling included integrins and extracellular matrix components, suggesting that TBC1D22B may influence the trafficking of cell‐adhesion molecules. These observations imply that TBC1D22B's function(s) might extend to include regulation of cell architecture and intercellular interactions. Furthermore, the combined analysis of the proximity and physical interactomes revealed clusters of metabolic enzymes, including those involved in *de novo* purine synthesis, carbohydrate metabolism, and glycolysis. These findings are consistent with previous reports linking TBC1D22B to metabolic activity in cancer cells^[^ ^10^ ^]^ and with developmental studies showing that in *Drosophila*, the TBC1D22B orthologue, dTBC1D22, regulates lipid droplet accumulation.^[^ ^27^ ^]^ Together these data suggest a possible role of TBC1D22B in coordinating trafficking with metabolic state, which might be subverted in cancer.

### 3.2 TBC1D22B Inhibits ER‐to‐Golgi Trafficking via RAB1B Inactivation

Functionally, TBC1D22B acts as a physiological inhibitor of ER‐to‐Golgi trafficking. This was demonstrated using the RUSH system, where overexpression of TBC1D22B delayed the Golgi arrival of a model cargo (GFP‐GPI), while TBC1D22B silencing accelerated it. The delay in trafficking was dependent on GAP activity, as the RQ mutant had no effect on ER‐to‐Golgi trafficking, despite retaining some post‐Golgi effects. This indicates that TBC1D22B's inhibition of anterograde transport is mediated through its GTPase activity.

Among the 19 RAB GTPases identified in the TBC1D22B interactomes, RAB1B emerged as the critical target. RAB1B knockdown recapitulated the trafficking delay induced by TBC1D22B overexpression, and direct GAP activity of TBC1D22B toward RAB1B was demonstrated in vitro. Furthermore, TBC1D22B preferentially bound GTP‐loaded RAB1B, consistent with its role in RAB inactivation.

While these results strongly support the notion that TBC1D22B functions as a RabGAP for RAB1B, they should be interpreted within the broader context of existing knowledge. RAB1B is primarily localized to the ER, ERES, and the Golgi.^[^ ^28^ ^]^ It is required for the transport of newly synthesized proteins from the ER to the Golgi, functioning through interactions with several effector molecules.^[^ ^29^ ^]^ At ERES, RAB1B binds to components of the COPII complex, modulating their interactions and promoting Golgi targeting of vesicles or elongated membrane tubules.^[^ ^29^, ^30^ ^]^

Current models indicate that RAB GTPases are activated by GEFs during vesicle budding and inactivated by GAPs at the fusion site.^[^ ^2^ ^]^ TBC1D22B might, therefore, be required to switch off RAB1B once it reaches the Golgi. Interestingly, a major GAP for RAB1B is TBC1D20, an ER‐resident GAP.^[^ ^14^, ^31^ ^]^ Thus, TBC1D22B and TBC1D20 may provide spatially distinct layers of RAB1B regulation – at the Golgi and ER, respectively – allowing for compartment‐specific tuning of vesicular trafficking.

The potential for TBC1D22B to regulate trafficking in a cargo‐specific manner remains an open and intriguing question. For instance, the TBC1D22A paralogue facilitates ER‐to‐Golgi transport of α2B‐adrenergic receptors, whereas TBC1D22B does not.^[^ ^15^ ^]^ This suggests a possible division of labor between paralogues, with TBC1D22B specializing in the retention or diversion of certain cargos – such as ECM proteins or metabolic enzymes – whose trafficking may influence cancer progression.

### 3.3 TBC1D22B Shapes Cancer Cell Behavior and Transcriptional Programs

The relevance of TBC1D22B's trafficking function to BC biology was confirmed in multiple experimental systems. Overexpression of TBC1D22B enhanced spheroid growth in 3D matrigel, a phenotype that is not replicated by either the RQ mutant or the TBC1D22A paralogue.

Transcriptomic profiling of TBC1D22B‐overexpressing BC cells revealed widespread gene expression changes, particularly transcriptional repression. Notably, a set of 159 genes was significantly downregulated both in BT549 cells overexpressing TBC1D22B and in TBC1D22B‐high Luminal BCs. This gene signature was highly enriched for secreted proteins involved in ECM organization, glycosaminoglycan binding, and cell adhesion – consistent with a trafficking‐based mechanism of transcriptional feedback or disruption of secretory homeostasis. The genes within this downregulated module exhibited strong co‐regulation across both the METABRIC and TCGA cohorts, suggesting that TBC1D22B overexpression leads to a reproducible, concerted repression of an ECM‐associated transcriptional program. Whether this is a direct consequence of trafficking perturbation or involves secondary signaling pathways remains unclear, but several downregulated transcription factors could mediate these effects.

### 3.4 Implications for Tumor Progression and Secretion Reprogramming

Our data also raise the possibility that TBC1D22B overexpression in tumors may facilitate a shift from canonical to unconventional secretory pathways. Inhibiting ER‐to‐Golgi transport could promote Golgi‐bypass routes of secretion – pathways increasingly recognized in cancer for releasing cytokines, enzymes, and glycoproteins that modulate the tumor microenvironment and promote metastasis.^[^ ^32^ ^]^ Thus, TBC1D22B may enable the selective release of pro‐tumorigenic factors via non‐canonical routes, reshaping the secretome in ways that benefit tumor progression.

It remains to be determined whether the effects of TBC1D22B on cancer‐relevant phenotypes are exclusively determined by its inactivation of RAB1B function. In addition to RAB1B, TBC1D22B may act on other RABs or perform GAP‐independent functions, as suggested by post‐Golgi transport delays observed with both TBC1D22B and the RQ mutant. RabGAPs are often promiscuous, and it is plausible that TBC1D22B participates in multiple trafficking steps, potentially coordinating a broader remodeling of cellular logistics in cancer cells.

Future studies are needed to delineate the full spectrum of TBC1D22B substrates and to characterize its impact on unconventional secretion and tumor microenvironment remodeling. Given its specificity and tumor‐promoting role, TBC1D22B represents a potential therapeutic target in BC, particularly in aggressive Luminal tumors.

## 4 Experimental Section

### Cell Cultures, Antibodies, and Reagents

BT549 cells, from ATCC, were grown in RPMI 1640 (ECB9006L, Euroclone) supplemented with 10% fetal bovine serum (ECS1800D, Euroclone), 1% L‐glutamine (ECB3000D, Euroclone), insulin (0.01 mg ml^−1^; 1882, Sigma) and 1% penicillin‐streptomycin (ECB3001D, Euroclone). CAL120 cells, from DSMZ, were grown in DMEM (ECM0749L, Euroclone) supplemented with 10% fetal bovine serum (ECS1800D, Euroclone), 1% L‐glutamine (ECB3000D, Euroclone) and 1% penicillin‐streptomycin (ECB3001D, Euroclone). Cells were authenticated by STR profiling (StemElite ID System, Promega) and periodically tested for mycoplasma with Venor GM Kit (56‐1010, Minerva Biolabs).

Antibodies and reagents were:

i) from Cell Signaling Technology: anti‐HA‐tag [C29F4 #3724, 1:1000 for immunoblotting (IB), 1:200 for immunofluorescence (IF)], anti‐GM130 (#12480 1:200 for IF).
iii) from Santa Cruz: anti‐ACBD3 (sc‐101277 1:1000 for IB), anti‐GAPDH (sc‐32233 1:1000 for IB), anti‐EGFR (sc‐03 1:200 for IB).
iv) from Abnova anti‐TBC1D22B (H00055633‐B01P, 1:200 for IF).
v) from Thermo Fisher Scientific Streptavidin‐HRP (VK318136 1:2000 for IB), Alexa Fluor 555‐Streptavidin (S21381, 1:400 for IF), Phalloidin 647 (A22287, 1:400 for IF of spheroids).
vi) from Sigma anti‐TBC1D22B (HPA027908 1:1000 for IB), anti‐vinculin (V9131 1:2000 for IB).
vii) from Novus anti‐TBC1D22A (NBP2‐88415 1:1000 for IB).

### Silencing Experiments and Engineering of Vectors

Transient silencing of TBC1D22B, TBC1D22A and the various RABs shown in Figures 4 and 5 was obtained by transfection of pools of 4 ON‐TARGETplus siRNA oligos (Dharmacon) (20 nM) for 96 h. Gene IDs and catalogue numbers of siRNA oligos used in the study are in Table S11 (Supporting Information). On day 1, cells were transfected with the siRNA oligos in suspension using Lipofectamine RNAiMAX (13778075, Invitrogen, Thermo Fisher Scientific), according to manufacturer's instructions. On day 2, a second round of transfection was performed on the adherent cells. On day 3 silenced cells were transfected with the RUSH construct (see RUSH experiments for details) and processed on day 5 (96 h after the first transfection). The APEX2‐TBC1D22B and APEX2‐TBC1D22B‐RQ constructs were engineered using Vector Builder with the following design: the Myc‐tagged APEX2 sequence was added in‐frame to the N‐terminus of the human HA‐tagged silencing‐resistant sequence of TBC1D22B, cloned into the lentiviral pLV vector. To generate the GAP‐defective TBC1D22B‐RQ mutant, the two catalytic residues, arginine 274 and glutamine 309, were substituted with alanine. In these constructs, two XhoI sites were inserted, one immediately after the Myc‐tag ATG start codon and the other immediately before the HA‐tag. Digestion with XhoI removed the Myc‐APEX2 sequence generating HA‐tagged TBC1D22B or TBC1D22B‐RQ in the pLV vector. The human, HA‐tagged, sequence of TBC1D22A was engineered by Vector Builder in the pLV vector. Puromycin‐resistant stable cell populations expressing these constructs were generated by infecting cells with viruses produced in HEK293T cells. mCherry‐tagged TBC1D22B and ‐RQ were engineered by Vector Builder in the pRP vector. Constructs encoding the glutathione S‐transferase (GST)‐tagged proteins were engineered by Vector Builder in the pET vector with a thrombin cleavage site between the GST sequence and the cDNA of human RAB1B, TBC1D22B or TBC1D22B‐RQ and transformed in BL21 inducible bacteria.

All constructs were sequence verified and were available upon request. The Str‐KDEL_SBP‐EGFP‐GPI construct was described in.^[^ ^19^ ^]^

### Real‐Time PCR

Total cellular RNA was extracted from silenced cells using the Maxwell RSC miRNA Tissue kit (AS1460, Promega) with the Maxwell RSC Instrument (AS4500, Promega). cDNA preparation was performed using the i‐Script reverse transcription Supermix Real Time qPCR (1708841, BioRAD) according to manufacturer's instructions, with the Veriti 96‐well Thermal Cycler (Applied Biosystems, Thermo Fisher Scientific). Quantitative Real‐Time PCR was performed on cDNA/reaction (30 ng) by applying the iTaq Universal SYBR Green Supermix (1725121 Biorad) and 500 nM of pre‐designed, Prime Time, primers from Integrated DNA Technologies.

### Proximity Biotinylation and Mass Spectrometry

BT549 cells stably expressing APEX2‐TBC1D22B, APEX2‐TBC1D22B‐RQ or empty vector (EV), were plated in 10‐cm dishes in quadruplicate (30 × 10^5^ cells/dish). After 24 h, two dishes per condition were incubated with biotin‐phenol (BP, 2 mM) (LS‐3500.0250 Iris Biotech GMBH) diluted in RPMI plus 10% FBS for 30 min at 37 °C. Next, cells were treated with 0.2 mM of H_2_O_2_ for 1 min at RT. Dishes were transferred immediately on ice and washed three times with the quencher solution (10 mM sodium ascorbate, 5 mM Trolox and 10 mM sodium azide solution in PBS). Cells were scraped in fresh quencher solution (1 mL per dish) and pelleted by centrifugation at 3000 g for 10 min at 4 °C. Pellets were lysed in ice‐cold lysis buffer (3 mL): RIPA (50 mM Tris, 150 mM NaCl, 0.5% SDS, 0.5%, sodium deoxycholate, 1% Triton X‐100), 1 M KCl, 3 M urea supplemented with protease inhibitors (P8340, Sigma‐Aldrich), 1 mM PMSF and quenchers (10 mM sodium ascorbate, 5 mM Trolox, 10 mM sodium azide). Lysates were clarified by centrifugation at 15 000 g for 30 min at 4 °C. Proteins were quantified with the Bradford method (5000001 Bio‐Rad.) Biotinylated proteins were pulled down with streptavidin magnetic beads (88816, Pierce, Thermo Fisher Scientific). In each condition, pulldowns were performed in triplicate, incubating lysate/sample (1.43 mg) with 50% slurry beads (90 µL) for 1 h at RT. Beads were washed twice with RIPA (1 mL), once with 1 M KCl (1 mL), four times with TBS (25 mM Tris, 150 mM NaCl; 1 mL) and once with 3 M urea (1 mL) in 50 mM NH_4_HCO_3_. After each washing step, beads were recovered using a magnetic stand. One sample was boiled in loading buffer (60 µL; NuPAGE LDS Sample Buffer NP0008 supplemented with NuPAGE Sample Reducing Agent, NP0009, Invitrogen) run on NuPAGE 4–12% Bis‐Tris Plus gel (NP0324BOX, Invitrogen) and stained with Colloidal Blue (LC6025, Thermo Fisher Scientific) to verify biotinylation and pulldown efficiency. The remaining two replicates were dried and frozen at ‐20 °C. The experiment was repeated twice obtaining a total of four replicates for mass spectrometry analysis.

Sample preparation for mass spectrometry was as follows: samples were resuspended in 50 mM NH_4_HCO_3_ and 3 M Urea. A reducing step was performed by adding 5 mM Tris(2‐carboxyethyl)phosphine (TCEP) and incubating the samples with orbital shaking for 1 h at 37 °C. Samples were then alkylated by adding 10 mM 2‐iodoacetamide in the dark with 20 min of orbital shaking at RT. Next, samples were supplemented with 20 mM DTT. Finally, buffer was exchanged with 50 mM NH_4_HCO_3_ and 2 M urea (300 µL). Each sample was digested with trypsin (1 µg; V5280, Promega, resuspended 1 µg µL^−1^ in 50 mM acetic acid) for 16 h at 37 °C. Supernatant was collected and beads were washed twice with 50 mM NH_4_HCO_3_ and 2 M urea (60 µL), pooling the washes with the supernatants. Acidification was achieved by adding 1% formic acid and samples were stored at −20 °C.

Tryptic peptides were suspended with 1% trifluoroacetic acid (5 µL) and analyzed by LC‐MS/MS using an EASY‐nLC 1200 (Thermo Fisher Scientific) coupled to a Q‐Exactive HF instrument (Thermo Fisher Scientific) through a nanoelectrospray ion source (EASY‐SPRAY, Thermo Fisher Scientific). The nano‐LC system was operated in one column set‐up with an EasySpray PEPMAP RSLC C18 column (Thermo Fisher Scientific) kept at 45 °C constant. Solvent A was 0.1% formic acid (FA) and solvent B was 0.1% FA in 80% acetonitrile. Samples were injected in aqueous 1% trifluoroacetic acid, at a constant pressure of 980 Bar. Peptides were separated with a gradient of 3–35% solvent B over 49 min followed by a gradient of 30–60% for 5 min and 60–95% over 5 min at a flow rate of 300 nL min^−1^. The Q‐Exactive was operated in the data‐dependent acquisition (DDA) to automatically switch between full scan MS and MSMS acquisition.

MS spectra (from m/z 375–1650) were analyzed in the Orbitrap detector with resolution R = 70 000 at m/z 400. The 12 most intense peptide ions with charge states ≥2 were sequentially isolated to a target value of 3e6 and fragmented with a normalized collision energy setting of 28% into the HCD cell. The maximum allowed ion accumulation times were 20 ms for full scans and 80 ms for MSMS. Acquired raw data obtained by mass spectrometry analysis were analyzed using the MaxQuant (MQ)^[^ ^33^ ^]^ version 1.6.10.43, and peptide lists were searched against the human Uniprot FASTA database (74470 Entries) with the Andromeda search engine.^[^ ^34^ ^]^

False discovery rate (FDR) for both protein and peptide identifications was set to a maximum of 1% with enzyme specificity set to Trypsin/P. A maximum of 2 missed cleavages was allowed, and the minimum peptide length was fixed at 7 amino acids and carbamidomethylation of Cysteine was specified as a fixed modification. Peptides were identified with an initial precursor mass deviation of 7 ppm and a fragment mass deviation of 20 ppm. For label‐free protein quantitation (LFQ), it required a minimum ratio count of 2.^[^ ^35^ ^]^ All proteins and peptides matching to the reversed database were filtered out. ProteinGroups.txt table from MQ output was analyzed using a homemade R Studio pipeline and a moderated t‐test statistics from an empirical Bayes method was used. In particular, to identify significantly regulated proteins between sample groups a Benjamini–Hochberg FDR correction corresponding to 5% together with a minimal fold‐change equal to 1 was imposed. Unless otherwise indicated, chemical powders were from Sigma. The mass spectrometry proteomics data have been deposited to the ProteomeXchange Consortium via the PRIDE^[^ ^36^ ^]^ partner repository with the dataset identifier PXD060457. Pathway analyses were done using STRING (https://string‐db.org/)^[^ ^18^ ^]^ and Enrich (https://maayanlab.cloud/Enrichr/).^[^ ^37^ ^]^

### Immunoprecipitation and Immunoblotting

In the co‐immunoprecipitation experiments of Figure S1F,G (Supporting Information), BT549 cells (1.5 × 10^6^) overexpressing HA‐TBC1D22B, HA‐TBC1D22B‐RQ or EV were plated in 10‐cm dishes 24 h before performing the experiment. The following day cells were washed three times with cold PBS and lysed in Pierce Buffer (25 mM Tris HCl pH 7.4, 150 mM NaCl, 1 mM EDTA pH 8, 1% NP‐40, 5% Glycerol; 500 µL) supplemented with 1X protease inhibitor cocktail, 2 mM PMSF, 50 mM NaF, 10 mM NaVO_4_, and 20 mM NaPPi. Lysates were kept on ice for 10 min, followed by 10 min centrifugation at 13 000 rpm at 4 °C. Proteins were quantified with the BCA method (23225, Pierce). Total lysates for each condition (2.5 mg) were incubated with anti‐HA Magnetic Beads (60 µL; 88836, Pierce), 50% slurry for 30 min at RT. Beads were then washed four times using Pierce Lysis Buffer (1 mL). After each washing step, beads were recovered using a magnetic stand and supernatant was removed. Samples were boiled at 96 °C for 10 min in loading buffer (60 µL), run on gels (Bolt 4–12% Bis‐Tris Plus; NW04120 or Bolt 8% Bis‐Tris Plus; NW00080, Invitrogen) and transferred to nitrocellulose membranes (1704158, BioRad).

In the co‐immunoprecipitation experiments subjected to the mass spectrometry (Figure 2), 8×10^6^ cells were plated in 15‐cm dishes 48 h before performing the experiment. Total lysates, obtained as above (1.75 mg/condition in quadruplicates), were incubated with anti‐HA Magnetic Beads (80 µL), slurry 50% for 1 h at RT. Beads were then washed twice using Pierce Lysis Buffer (1 mL) and once with H_2_O (1 mL). After each washing step, beads were recovered using a magnetic stand and supernatant was removed. Protein complexes were eluted in glycine 0.1 M pH 2 (100 µL) shaking at RT for 15 min. Neutralization was achieved by adding TRIS‐HCl 1 M pH 8.0 (20 µL).

### Immunofluorescence

BT549 cells were fixed in paraformaldehyde (PFA) 4% for 10 min at RT and permeabilized in 0.1% Triton X‐100 in PBS for 10 min at RT. Incubation with primary and secondary antibodies was performed in PBS BSA 1% for 1 h at RT. Coverslips were washed three times in PBS, rinsed in H_2_O and mounted with Fluoromount‐G. Images were acquired using a Leica SP8 AOBS confocal microscope and analyzed with ImageJ. In the experiments evaluating the biotinylating activity of the APEX2 chimeras by immunofluorescence, 1.0 × 10^5^ BT549 cells expressing APEX2‐TBC1D22B or APEX2‐TBC1D22B‐RQ were plated in a six‐well plate on coverslips coated with 0.5% Gelatin. After 48 h, cells were treated with 500 µM of biotin‐phenol diluted in RPMI 10% FBS (2 mL per well) for 30 min at 37 °C. Next, cells were treated with 1 mM H_2_O_2_ for 1 min at RT. Cells were transferred on ice and washed three times with quencher solution, fixed in PFA 4% for 10 min at RT and processed as described above. Strepatividin‐555 (1:1000 v/v in PBS, BSA 1%) was added together with the secondary antibody for 30 min at RT.

### RUSH Experiments

In the RUSH experiments shown in Figure 3 and Figure S3 (Supporting Information), BT549 and CAL120 cells (respectively) expressing the empty pLV vector (EV), HA‐TBC1D22B‐WT or HA‐TBC1D22B‐RQ (1.0 × 10^5^) were seeded in six‐well plates on coverslips coated with 0.5% gelatin. Twenty‐four h later, coverslips were transferred in 24 multi‐well plates and cells were transfected with the Str‐KDEL_SBP‐EGFP‐GPI construct. In the RUSH experiments shown in Figures 4 and 5, silenced cells were transiently transfected with the Str‐KDEL_SBP‐EGFP‐GPI construct 24 h after the second round of transfection with the siRNA oligos. Transfection of the Str‐KDEL_SBP‐EGFP‐GPI construct was performed by preparing a mixture containing DNA (0.5 µg), X‐tremeGENE DNA Transfection Reagent (1 µL; Roche), and Opti‐MEM medium (100 µL), which was incubated at RT for 15 min before adding to the cells. Cells were kept in DMEM, 10% FBS, 0.1 µM avidin during transfection. Forty‐eight h later, cells were washed twice with PBS, followed by 15 min at 37 °C in DMEM 10% FBS. Next, 40 µM biotin was added to the medium to release the reporter. Coverslips were fixed at different time points and processed for immunofluorescence. We observed that, in BT549 control cells, the relocalization of the GFP‐GPI reporter to the Golgi was maximal 20 min after biotin addition. For this reason, in the experiments done on BT549 cells silenced with control oligos or with oligos for TBC1D22B or TBC1D22A, an additional time point 8 min was added between time 0 and time 20 min to better appreciate the acceleration of GFP‐GPI re‐localization to the Golgi in the silenced cells.

For live cell imaging experiments, BT549 cells were seeded on 25 mm coverslips and co‐transfected, as described above, with the Str‐KDEL_SBP‐EGFP‐GPI construct alone or together with mCherry‐TBC1D22B or mCherry‐TBC1D22B‐RQ. Coverslips were put on L‐shaped Chamlide assembled with tubing and syringes to replace DMEM 10% 0.1 µM avidin with Lebovitz medium containing 80 µM of biotin. Images were captured using the Inverted Eclipse Ti‐E (Nikon)+ Spinning Disk CSU‐X1 (Yokogawa Integrated in Metamorph software by Gataca System), equipped with a 60x CFI plan Apo VC objective, GFP, mCherry filters and an iXon Ultra 897 (Andor) EMCCD camera.

Quantification of the GFP‐GPI staining on fixed cells was performed using a macro developed through ImageJ software (version 1.54f). For each individual cell, the GM130 fluorescence signal was used to create a mask corresponding to the Golgi apparatus. The GFP‐GPI signal within the mask was quantified and used to calculate the percentage of GFP‐GPI signal present in the Golgi relative to the GFP‐GPI signal measured in the entire cell.

### Spheroid Growth

Cells were seeded in 3D matrices to assess spheroid formation and monitor their growth over time. On day 1, matrigel Geltrex LDEV‐free (60 µl; A14132‐02, Gibco) were added per well into 8‐well chambered polystyrene slides (CLS354118 tissue culture‐treated glass, Falcon) and incubated for 20 min at RT. Subsequently, cells were seeded in culture medium (150 µL) and incubated for 30 min at RT. Additional medium containing 4% matrigel (150 µL) was then added to each well. Spheroids were cultured for 7 days, with medium replacement every 2–3 days. Their formation was monitored from day 3 to day 7 using a Leica DMI3000B optical microscope. On day 7, spheroids were washed with PBS, fixed in 4% paraformaldehyde for 30 min at RT, and permeabilized using 0.5% Triton X‐100 in PBS for 15 min at RT. They were then washed with PBS containing 300 nM glycine for 10 min. For blocking, spheroids were incubated for 1.5 h at RT in IF buffer (PBS with 0.1% BSA, 0.2% Triton X‐100, and 0.05% Tween‐20) supplemented with 5% donkey serum. After three washes in IF buffer, they were incubated for 1.5 h with Phalloidin and DAPI diluted in the same buffer with 5% donkey serum. Following two additional washes in IF buffer and one in PBS, spheroids were mounted on glass slides using Fluormount‐G. Imaging was performed with a Leica SPE confocal microscope (Leica Microsystems), using 20x or 40x objectives and acquiring Z‐stacks (8 optical sections per image).

### Protein Production

BL21 bacteria expressing GST‐RAB1B were grown overnight at 37 °C in 100 ml of LB in presence of 100 µg ml^−1^ ampicillin. The day after, bacteria were diluted 1:100 in 1 L of LB and grown until OD600 = 0.6, then induced with 1 mM IPTG for 5 h. Bacteria were spun at 3000 rpm for 15 min at 4 °C, lysed in Lysis Buffer (50 mM TrisHCl pH 8.0, 100 mM NaCl pH 8.2 mM MgCl_2_, 0.1% β‐mercaptoethanol plus protease inhibitors and 10 µg ml^−1^ PMSF) and frozen at ‐20 °C. The day after they were thawed, lysozyme was added to a final concentration of 0.02 mg ml^−1^. The bacterial lysate was sonicated (amplitude 30%, 20 sec followed by 10 sec pause for three times) supplemented with 1% Triton‐X100 and centrifuged at 14 000 rpm for 1 h at 4 °C. The supernatant was applied to glutathione (GSH) magnetic beads (500 µl 50% slurry cat. N. 78602, Invitrogen) for 1 h at 4 °C. Beads were washed three times with Lysis Buffer supplemented with 1% TritonX‐100 and three times with Lysis Buffer. Beads were resuspended in Lysis Buffer (250 µL). To quantify protein yield, 10 µl and 20 µl of slurry beads were taken and eluted by boiling for 10 min at 96 °C in Loading Buffer. Samples were resolved by SDS‐PAGE and stained with Colloidal blue together with known quantities of BSA for protein concentration estimation.

The protocol was slightly modified to produce GST‐TBC1D22B and GST‐TBC1D22B‐RQ. In this case, the overnight bacterial culture was diluted 1:100 and 2 L per construct were prepared. After reaching an OD600 = 1.6, BL21 bacteria were induced overnight with 0.5 mM IPTG. The bacterial pellet was resuspended in Lysis buffer (40 mL; 50 mM TrisHCl pH 8.0, 100 mM NaCl, 2 mM MgCl_2_, 0.1% β‐mercaptoethanol plus protease inhibitors and 10 µg ml^−1^ PMSF) and incubated with 50% slurry glutathione agarose beads (500 µL; 16100, Pierce) previously washed in Lysis Buffer. Purified proteins on beads were washed two times GAP Buffer (1 mL; 20 mM HEPES pH 7.5, 150 mM NaCl, 11 mM MgCl_2_) and resuspended in GAP Buffer (100 µL). GST‐TBC1D22B and GST‐TBC1D22B‐RQ were cleaved from the GST moiety by adding biotinylated thrombin (1 µL, 0.5U µL^−1^) for 2 h at RT. The excess of thrombin was removed adding streptavidin‐coated magnetic beads (10 µL; 88816, Pierce) for 30 min at RT.

### GAP Assays

GAP assays were performed using the Malachite green phosphate assay (MAK‐307, Sigma‐Aldrich). GAP assays were performed in triplicate using TBC1D22B, TBC1D22B‐RQ and GAP buffer alone as a negative control. GST‐RAB1B (200 pmol) and TBC1D22B or TBC1D22B‐RQ (10 pmol) were used per reaction (ratio of 20:1). GST‐RAB1B (on magnetic GSH beads) was loaded with 60 nmol of GTP in GTP‐Loading Buffer (1 mL; 20 mM HEPES pH 7.5, 150 mM NaCl, 5 mM EDTA, 1 mM DTT) for 20 min at 30 °C, then 1 M MgCl_2_ was added for an additional 5 min at 30 °C. Beads were recovered on a magnetic stand and washed with GAP buffer (1 mL; 20 mM HEPES pH 7.5, 150 mM NaCl, 11 mM MgCl_2_). TBC1D22B or TBC1D22B‐RQ or GAP buffer (negative control) were added to a final volume of 100 µL and incubated for 8 min at 30 °C. Aliquots (25 µL) in triplicates were used for the measurements and transferred to a 96 multi‐well plate. Malachite green reaction mix was added, and the colorimetric reaction was read 30 min later using the Tecan Spark10M spectrophotometer. The amount of phosphate released was calculated from a standard curve according to the manufacturer's instruction.

### Pull Down Assays

Ten µg of purified GST‐RAB1B (on magnetic GSH beads) were washed twice with buffer A (50 mM Hepes pH 7.5, 100 mM NaCl, 5 mM EDTA) and incubated in buffer A for 30 min at 4 °C rocking. After two further washes in buffer A, GST‐RAB1B was loaded with either 1 mM GTP‐γ‐S (G8634, Merck) or GDP (G7127, Merck) in 100 µl of loading buffer (20 mM Hepes pH 7.5, 50 mM NaCl, 1 mM DTT) for 30 min at 30 °C. Next, 10 mM MgCl_2_ was added to the samples and incubated for additional 10 min at 30 °C.

Total cellular lysates were prepared from 70% confluent BT549 cells stably infected with EV, HA‐TBC1D22B or HA‐TBC1D22A. Cells were washed twice with ice‐cold PBS and lysed by scraping in ice‐cold lysis buffer (50 mM HEPES, 150 mM NaCl, 10% glycerol, 1% TritonX‐100, 5 mM EGTA, 1.5 mM MgCl_2_, proteases and phosphatases inhibitor cocktail). Lysates were cleared by spinning at 13 000 rpm for 30 min 4 °C. After quantification with the BCA method, total lysates (500 µg) were applied to GST‐RAB1B (10 µg) loaded with either GTP‐γ‐S or GDP, and to GST as negative control, and incubated at 4 °C for 1 h rocking. Beads were recovered with the magnetic stand and washed four times with ice‐cold lysis buffer. Samples were boiled at 96 °C for 10 min in loading buffer (30 µL) and 1/3 of the pull down (10 µL) was run on gels (NuPAGE 4–12% Invitrogen) and transferred to nitrocellulose membranes.

### RNAseq

For RNAseq, total cellular RNA was extracted, and the quality was assessed using the Bioanalyzer 2100 (Agilent). Total RNA was depleted of ribosomal RNA and the RNAseq libraries were prepared with the Illumina TruSeq Stranded Total RNA kit. Following adapter ligation, libraries were amplified by PCR, checked on a Bioanalyzer 2100, quantified with picogreen reagent (Invitrogen), and sequenced for 100 bases in the paired‐end mode with 50 million reads coverage on a Novaseq 6000 sequencer. Raw data were acquired for all datasets, and the human reference genome (hg38) was employed as the alignment template for mapping the reads through Bowtie2 (version 2.4.5). Following RNAseq, RNA counts were measured using the RESM software (version 1.3.3), and the unprocessed data were brought into the EdgeR package within the R software (version 3.40.2). Using default parameters, after filtering for not expressed or low expressed genes, library sizes were normalized and statistical analyses between groups were performed with the quasi‐likelihood F‐tests (QLF). Differentially expressed genes were obtained and the p‐value adjusted with the Benjamini and Hochberg methodology to obtain the FDR (false discovery rate). The complete set of data used for the analyses is provided in Table S12 (Supporting Information).

### Statistical Analyses

Statistical methods used to analyze the mass spectrometry data were detailed in the Proximity Biotinylation and Mass Spectrometry paragraph. Statistical analyses for Figures 3B, 4C, 5B, 6B, 7A‐B, and Figures S3B and S4A (Supporting Information) were performed by two‐tailed unpaired *t*‐test, assuming normal distributions. Statistical analyses for Figures 3C, 4D, 5C, and Figure S3C (Supporting Information) were performed with the Fisher's test.

## Conflict of Interest

The authors declare no conflict of interest

## Author Contributions

F.M., M.L., and A.M. equally contributed to the work and are co‐first authors. F.M. conducted most of the experiments of the original submission. M.L. performed experiments in the original submission and the RUSH assays in the revised version. A.M. performed the spheroids formation assays and participated in many experiments of the revised version. F.B. (Fabio Bedin) performed the proteomic bioinformatic analyses in the original submission. G.V., L.A., B.M., R.P., participated in many experiments of the original submission (G.V., B.M.) or revised version (L.A., R.P.). S.F. (Stefano Freddi) quantified the RUSH experiments. S.F. (Stella Fontana) did pull down assays for the revised version. A.F. performed the transcriptomic bioinformatic analyses. G.B. supported F.M. in the RUSH experiments. F.P. provided theoretical guidance in the RUSH assays. F.B. (Federico Bussolino) provided financial support and technical guidance. A.C. provided theoretical guidance and analyzed the proteomic experiments. S.S. helped in analyzing data and writing the article. L.L. designed the study, analyzed data, provided financial and theoretical guidance and wrote and review the article. All authors critically reviewed the manuscript.

## Supporting information



Supporting Information

Supplemental Movie 1

Supplemental Movie 2

Supplemental Movie 3

Supporting Information

Supporting Information

Supporting Information

Supporting Information

Supporting Information

Supporting Information

## Data Availability

The data that support the findings of this study are available from the corresponding author upon reasonable request.

## References

[1] a) A. Zeigerer , R. L. Bogorad , K. Sharma , J. Gilleron , S. Seifert , S. Sales , N. Berndt , S. Bulik , G. Marsico , R. C. J. D'Souza , N. Lakshmanaperumal , K. Meganathan , K. Natarajan , A. Sachinidis , A. Dahl , H.‐G. Holzhütter , A. Shevchenko , M. Mann , V. Koteliansky , M. Zerial , Cell Rep. 2015, 11, 884; 25937276 10.1016/j.celrep.2015.04.018

[2] M. A. Frasa , K. T. Koessmeier , M. R. Ahmadian , V. M. Braga , Nat. Rev. Mol. Cell Biol. 2012, 13, 67. 22251903 10.1038/nrm3267

[3] X. Pan , S. Eathiraj , M. Munson , D. G. Lambright , Nature 2006, 442, 303. 16855591 10.1038/nature04847

[4] a) W. G. Roach , J. A. Chavez , C. P. Miinea , G. E. Lienhard , Biochem. J. 2007, 403, 353; 17274760 10.1042/BJ20061798 PMC1874243

[5] a) M. Kitano , M. Nakaya , T. Nakamura , S. Nagata , M. Matsuda , Nature 2008, 453, 241; 18385674 10.1038/nature06857

[6] a) M. E. Oguchi , K. Noguchi , M. Fukuda , PLoS One 2017, 12, 0174883; 10.1371/journal.pone.0174883 PMC5383037 28384198

[7] R. M. Nottingham , G. V. Pusapati , I. G. Ganley , F. A. Barr , D. G. Lambright , S. R. Pfeffer , J. Biol. Chem. 2012, 287, 22740. 22637480 10.1074/jbc.M112.362558 PMC3391118

[8] E. Frittoli , A. Palamidessi , A. Pizzigoni , L. Lanzetti , M. Garrè , F. Troglio , A. Troilo , M. Fukuda , P. P. Di Fiore , G. Scita , S. Confalonieri , Mol. Biol. Cell 2008, 19, 1304. 18199687 10.1091/mbc.E07-06-0594 PMC2291429

[9] S. Sigismund , L. Lanzetti , G. Scita , P. P. Di Fiore , Nat. Rev. Mol. Cell Biol. 2021, 22, 625. 34075221 10.1038/s41580-021-00375-5

[10] M. Lupi , D. Avanzato , S. Confalonieri , F. Martino , R. Pennisi , E. Pupo , V. Audrito , S. Freddi , G. Bertalot , F. Montani , B. Matoskova , S. Sigismund , P. P. Di Fiore , L. Lanzetti , Cell Death Dis. 2024, 15, 647. 39231952 10.1038/s41419-024-07037-2 PMC11375060

[11] H. Pan , R. Gray , J. Braybrooke , C. Davies , C. Taylor , P. McGale , R. Peto , K. I. Pritchard , J. Bergh , M. Dowsett , D. F. Hayes , N. Engl. J. Med. 2017, 377, 1836. 29117498 10.1056/NEJMoa1701830 PMC5734609

[12] a) X. Yue , M. Bao , R. Christiano , S. Li , J. Mei , L. Zhu , F. Mao , Q. Yue , P. Zhang , S. Jing , J. E. Rothman , Y. Qian , I. Lee , FEBS Lett. 2017, 591, 2793; 28777890 10.1002/1873-3468.12780

[13] Y. Huang , L. Yang , Y.‐Y. Pei , J. Wang , H. Wu , J. Yuan , L. Wang , Exp. Cell Res. 2018, 363, 39. 29307786 10.1016/j.yexcr.2018.01.003

[14] A. K. Haas , S. Yoshimura , D. J. Stephens , C. Preisinger , E. Fuchs , F. A. Barr , J. Cell Sci. 2007, 120, 2997. 17684057 10.1242/jcs.014225

[15] Z. Wei , M. Zhang , C. Li , W. Huang , Y. Fan , J. Guo , M. Khater , M. Fukuda , Z. Dong , G. Hu , G. Wu , Cell Rep. 2019, 28, 554. 31291588 10.1016/j.celrep.2019.05.033 PMC6639060

[16] V. Hung , N. D. Udeshi , S. S. Lam , K. H. Loh , K. J. Cox , K. Pedram , S. A. Carr , A. Y. Ting , Nat. Protoc. 2016, 11, 456. 26866790 10.1038/nprot.2016.018 PMC4863649

[17] D. Mellacheruvu , Z. Wright , A. L. Couzens , J.‐P. Lambert , N. A. St‐Denis , T. Li , Y. V. Miteva , S. Hauri , M. E. Sardiu , T. Y. Low , V. A. Halim , R. D. Bagshaw , N. C. Hubner , A. al‐Hakim , A. Bouchard , D. Faubert , D. Fermin , W. H. Dunham , M. Goudreault , Z.‐Y. Lin , B. G. Badillo , T. Pawson , D. Durocher , B. Coulombe , R. Aebersold , G. Superti‐Furga , J. Colinge , A. J. R. Heck , H. Choi , M. Gstaiger , et al., Nat. Methods 2013, 10, 730. 23921808 10.1038/nmeth.2557 PMC3773500

[18] D. Szklarczyk , R. Kirsch , M. Koutrouli , K. Nastou , F. Mehryary , R. Hachilif , A. L. Gable , T. Fang , N. T. Doncheva , S. Pyysalo , P. Bork , L. J. Jensen , C. v. Mering , Nucleic Acids Res. 2023, 51, D638. 36370105 10.1093/nar/gkac1000 PMC9825434

[19] G. Boncompain , S. Divoux , N. Gareil , H. de Forges , A. Lescure , L. Latreche , V. Mercanti , F. Jollivet , G. Raposo , F. Perez , Nat. Methods 2012, 9, 493. 22406856 10.1038/nmeth.1928

[20] a) E. A. Miller , R. Schekman , Curr. Opin. Cell Biol. 2013, 25, 420; 23702145 10.1016/j.ceb.2013.04.005 PMC3736695

[21] S. Munro , Cold Spring Harb Perspect Biol 2011, 3, a005256. 21436057 10.1101/cshperspect.a005256 PMC3098672

[22] T. Weide , M. Bayer , M. Koster , J. P. Siebrasse , R. Peters , A. Barnekow , EMBO Rep. 2001, 2, 336. 11306556 10.1093/embo-reports/kve065 PMC1083862

[23] R. Valsdottir , H. Hashimoto , K. Ashman , T. Koda , B. Storrie , T. Nilsson , FEBS Lett. 2001, 508, 201. 11718716 10.1016/s0014-5793(01)02993-3

[24] a) M. Wong , S. Munro , Science 2014, 346, 1256898; 25359980 10.1126/science.1256898 PMC4254398

[25] S. W. Hicks , T. A. Horn , J. M. McCaffery , D. M. Zuckerman , C. E. Machamer , Traffic 2006, 7, 1666. 17118120 10.1111/j.1600-0854.2006.00504.x

[26] I. Raote , M. Ortega‐Bellido , A. J. Santos , O. Foresti , C. Zhang , M. F. Garcia‐Parajo , F. Campelo , V. Malhotra , Elife 2018, 7, 32723. 10.7554/eLife.32723 PMC5851698 29513218

[27] X. Duan , L. Xu , Y. Li , L. Jia , W. Liu , W. Shao , V. Bayat , W. Shang , L. Wang , J.‐P. Liu , C. Tong , Cell Rep. 2021, 36, 109541. 34469730 10.1016/j.celrep.2021.109541

[28] H. Martinez , I. A. Garcia , L. Sampieri , C. Alvarez , PLoS One 2016, 11, 0160838. 10.1371/journal.pone.0160838 PMC4976911 27500526

[29] a) H. Plutner , A. D. Cox , S. Pind , R. Khosravi‐Far , J. R. Bourne , R. Schwaninger , C. J. Der , W. E. Balch , J. Cell Biol. 1991, 115, 31; 1918138 10.1083/jcb.115.1.31 PMC2289927

[30] Y. Hirata , Y. Matsui , I. Wada , N. Hosokawa , Mol. Biol. Cell 2022, 33, ar21. 35044867 10.1091/mbc.E21-07-0372 PMC9250382

[31] E. H. Sklan , R. L. Serrano , S. Einav , S. R. Pfeffer , D. G. Lambright , J. S. Glenn , J. Biol. Chem. 2007, 282, 36354. 17901050 10.1074/jbc.M705221200

[32] S. Vats , T. Galli , Front Cell Dev Biol 2022, 10, 884020. 35784483 10.3389/fcell.2022.884020 PMC9244844

[33] S. Tyanova , T. Temu , J. Cox , Nat. Protoc. 2016, 11, 2301. 27809316 10.1038/nprot.2016.136

[34] J. Cox , N. Neuhauser , A. Michalski , R. A. Scheltema , J. V. Olsen , M. Mann , J. Proteome Res. 2011, 10, 1794. 21254760 10.1021/pr101065j

[35] J. Cox , M. Y. Hein , C. A. Luber , I. Paron , N. Nagaraj , M. Mann , Mol. Cell. Proteomics 2014, 13, 2513. 24942700 10.1074/mcp.M113.031591 PMC4159666

[36] Y. Perez‐Riverol , C. Bandla , D. J. Kundu , S. Kamatchinathan , J. Bai , S. Hewapathirana , N. S. John , A. Prakash , M. Walzer , S. Wang , J. A. Vizcaíno , Nucleic Acids Res. 2025, 53, D543. 39494541 10.1093/nar/gkae1011 PMC11701690

[37] M. V. Kuleshov , M. R. Jones , A. D. Rouillard , N. F. Fernandez , Q. Duan , Z. Wang , S. Koplev , S. L. Jenkins , K. M. Jagodnik , A. Lachmann , M. G. McDermott , C. D. Monteiro , G. W. Gundersen , A. Ma'ayan , Nucleic Acids Res. 2016, 44, W90. 27141961 10.1093/nar/gkw377 PMC4987924

