# Supplementary material for: TBC1D22B Regulates ER‐to‐Golgi Trafficking via RAB1B Inactivation and Promotes Oncogenic Programs in Breast Cancer
Source: Adv Sci (Weinh). 2025 Aug 29;12(43):e02269. doi: 10.1002/advs.202502269 (PMC12631847; doi:10.1002/advs.202502269)
Supplement: Supplementary file 1 — Supporting Information [file ADVS-12-e02269-s010.docx]

**Supporting Information**

**TBC1D22B Regulates ER-to-Golgi Trafficking via RAB1B Inactivation and Promotes Oncogenic Programs in Breast Cancer**

*Flavia Martino, Mariadomenica Lupi, Alessandra Murabito, Fabio Bedin, Giulia Villari, Linda Andreoli, Stefano Freddi, Bronislava Matoskova, Rosa Pennisi, Stella Fontana, Amir Fardin, Gaelle Boncompain, Franck Perez, Federico Bussolino,* *Alessandro Cuomo, Sara Sigismund and Letizia Lanzetti* *

F. Martino, M. Lupi, G.Villari, R. Pennisi, S. Fontana, F. Bussolino, L. Lanzetti

Department of Oncology, University of Torino Medical School, Torino, Italy.

Candiolo Cancer Institute, FPO - IRCCS, Candiolo, Torino, Italy.

E-mail: letizia.lanzetti@ircc.it

A. Murabito

Candiolo Cancer Institute, FPO - IRCCS, Candiolo, Torino, Italy.

F. Bedin

San Raffaele Telethon Institute for Gene Therapy (SR-Tiget), IRCCS San Raffaele Scientific Institute, Milan, Italy.

L. Andreoli, S. Freddi, B. Matoskova, A. Fardin, A. Cuomo, S. Sigismund

IEO, European Institute of Oncology IRCCS, Milan, Italy

S. Freddi, S. Sigismund

Department of Oncology and Hematology-Oncology, University of Milan, Milan Italy

G. Boncompain, F. Perez

Institut Curie, PSL Research University, Sorbonne Université, Centre National de la Recherche Scientifique, CNRS UMR144, Paris, France.

G. Boncompain present address

Institut Neuromyogene, CNRS/UCBL UMR5261, INSERM U1315, Université Claude Bernard Lyon1, Lyon, France

F. Martino, M. Lupi, A. Murabito contributed equally.

**SUPPLEMENTARY TABLES**

**Table S1. Proteins identified in the TBC1D22B proximity-labeling experiments**

The four sheets display all the hits identified by mass spectrometry in the following comparisons: TBC1D22B (henceforth 22B) vs. EV (sheet 1), 22B vs. H₂O₂ (sheet 2), RQ vs. EV (sheet 3), and RQ vs. H₂O₂ (sheet 4). The table has been filtered directly from the output of MaxQuant, ensuring consistency with the original dataset. Gene names were extracted directly from the human UniProt FASTA database (74,470 entries), searched using the Andromeda search engine.^[1]^ In some cases, gene names do not conform to the HUGO nomenclature (however, HUGO-compliant names are consistently used in the detailed analyses presented in Table S2). For certain genes, the notation “.1” is appended to the gene name when two protein IDs correspond to the same gene, to avoid redundancy. The label-free quantification (LFQ) values for each condition and replicate are reported. These values have been log-transformed, and the table is not imputed; where MaxQuant did not assign a value, the cell is marked as NaN (not a number). Additionally, the number of peptides identified per protein in the 22B or RQ replicates, as well as the protein name and gene name, are provided for all conditions.

Data are in Supplementary Table 1.xlsx

**Table S2. Details of the TBC1D22B proximity-labeling analysis**

**Sheet 1 (Significant 22B *vs*. EV).** Proteins significantly enriched in the 22B *vs*. EV comparison are reported. For each entry, we show: Gene Name (HUGO); Protein IDs; Protein Name; Fold change in log_2_ (22B/EV); p.adj, t-test adjusted with the Benjamini-Hochberg correction (in log_10_); p.val, t-test in log_10_; LFQ, label-free quantitation (in log_2_) for 4 replicates of 22B (22B1-4) and 4 replicates of EV (EV1-4); Pep, number of peptides identified/protein in 4 replicates of 22B (22B1-4); Peptide filter +, indicating that at least 2 peptides/protein were identified in all 4 22B replicates.

**Sheet 2 (Significant 22B *vs*. H_2_O_2_).** Proteins significantly enriched in the 22B *vs*. H_2_O_2_ comparison are reported. Categories are as in sheet 1 (herein H_2_O_2_ substitutes for EV).

**Sheet 3 (Significant 22B *vs*. EV-H_2_O_2_).** The list of 93 proteins (indicated by the HUGO gene name) significantly enriched in the 22B *vs*. EV/H_2_O_2_ comparison is shown. The Venn diagram shows the overlap between the significantly enriched proteins in the 22B *vs*. EV and 22B *vs*. H_2_O_2_ comparisons.

**Sheet 4 (Unique 22B *vs*. EV).** Proteins unique in the 22B *vs*. EV comparison are reported. For each entry, we show: Gene Name (HUGO); Protein IDs; Protein Name; LFQ, label-free quantitation (in log_2_) for 4 replicates of 22B (22B 1-4) and 4 replicates of EV (EV1-4); Pep, number of peptides identified/protein in 4 replicates of 22B (22B 1-4); Peptide filter +, indicating that at least 2 peptides/protein were identified in all 4 22B replicates.

**Sheet 5 (Unique 22B *vs*. H_2_O_2_).** Proteins unique in the 22B *vs*. H_2_O_2_ comparison are reported. Categories are as in sheet 4 (herein H_2_O_2_ substitutes for EV).

**Sheet 6 (Unique 22B *vs*. EV-H_2_O_2_).** The list of 333 proteins (HUGO gene name) unique in the 22B *vs*. EV/H_2_O_2_ comparison is shown. The Venn diagram shows the overlap between the unique proteins in the 22B *vs*. EV and 22B *vs*. H_2_O_2_ comparisons.

**Sheet 7 (Significant RQ *vs*. EV).** Proteins significantly enriched in the RQ *vs*. EV comparison are reported. For each entry, we show: Gene Name (HUGO); Protein IDs; Protein Name; Fold change in log_2_ (RQ/EV); p.adj, t-test adjusted with the Benjamini-Hochberg correction (in log_10_); p.val, t-test in log_10_; LFQ, label-free quantitation (in log_2_) for 4 replicates of RQ (RQ1-4) and 4 replicates of EV (EV1-4); Pep, number of peptides identified/protein in 4 replicates of RQ (RQ1-4); Peptide filter +, indicating that at least 2 peptides/protein were identified in all 4 RQ replicates.

**Sheet 8 (Significant RQ *vs*. H_2_O_2_).** Proteins significantly enriched in the RQ *vs*. H_2_O_2_ comparison are reported. Categories are as in sheet 7 (herein H_2_O_2_ substitutes for EV).

**Sheet 9 (Significant RQ *vs*. EV-H_2_O_2_).** The list of 107 proteins (HUGO gene name) significantly enriched in the RQ *vs*. EV/H_2_O_2_ comparison is shown. The Venn diagram shows the overlap between the significantly enriched proteins in the RQ *vs*. EV and RQ *vs*. H_2_O_2_ comparisons.

**Sheet 10 (Unique RQ *vs*. EV).** Proteins unique in the RQ *vs*. EV comparison are reported. For each entry, we show: Gene Name (HUGO); Protein IDs; Protein Name; LFQ, label-free quantitation (in log_2_) for 4 replicates of RQ (RQ1-4) and 4 replicates of EV (EV1-4); Pep, number of peptides identified/protein in 4 replicates of RQ (RQ1-4); Peptide filter +, indicating that at least 2 peptides/protein were identified in all 4 RQ replicates.

**Sheet 11 (Unique RQ *vs*. H_2_O_2_).** Proteins unique in the RQ *vs*. H_2_O_2_ comparison are reported. Categories are as in sheet 10 (herein H_2_O_2_ substitutes for EV).

**Sheet 12 (Unique RQ *vs*. EV-H_2_O_2_).** The list of 330 proteins (HUGO gene name) unique in the RQ *vs*. EV/H_2_O_2_ comparison is shown. The Venn diagram shows the overlap between the unique proteins in the RQ *vs*. EV and RQ *vs*. H_2_O_2_ comparisons.

**Sheet 13 (Crapome analysis). A.** The four lists of proteins (HUGO gene name) are shown. In red, proteins discarded after application of the Crapome filter (cut-off 25%, see main text for details) are shown. **B.** Proteins remaining after the crapome filter are shown.

**Sheet 14 (Overlap**). Lists of proteins (HUGO gene name) and Venn diagrams, derived from the overlap of unique proteins using the stringent and the less stringent filters (see main text for details).

**Sheet 15 (GO terms**). The complete list of GO terms, obtained by Enrich (https://maayanlab.cloud/Enrichr/), enriched in the group of 418 “unique” proteins at an FDR < 0.05.

Data are in Supplementary Table 2.xlsx

**Table S3. Total identified proteins in the TBC1D22B co-IP interactome**

The table shows all the hits identified by mass spec in the comparison 22B *vs*. EV (sheet 1). Gene names are reported as obtained directly from the human Uniprot FASTA database (74470 Entries) searched with the Andromeda search engine ^[1]^ and, in some cases, they do not follow the HUGO nomenclature (HUGO nomenclature is however used in all detailed analyses presented in Table S4). In some cases, genes are marked with the notation “.1”. This was done when two protein IDs referred to the same gene name, to avoid redundancy. The LQF (label-free quantitation) of each condition and each replicate is reported, together with the number of peptides identified/proteins in 22B replicates.

Data are in Supplementary Table 3.xlsx

**Table S4. Details of the analysis of the TBC1D22B co-IP interactome**

**Sheet 1 (Significant 22B *vs*. EV).** Proteins significantly enriched in the 22B *vs*. EV comparison are reported. For each entry, we show: Gene Name (HUGO); Protein IDs; Protein Name; Fold change in log_2_ (22B/EV); p.adj, t-test adjusted with the Benjamini-Hochberg correction (in log_10_); p.val, t-test in log_10_; LFQ, label-free quantitation (in log2) for 4 replicates of 22B (22B 1-4) and 4 replicates of EV (EV1-4); Pep, number of peptides identified/protein in 4 replicates of 22B (22B1-4); Peptide filter +, indicating that at least 2 peptides/protein were identified in all 4 22B replicates.

**Sheet 2 (Unique 22B *vs*. EV).** Proteins unique in the 22B *vs*. EV comparison are reported. Categories are as in sheet 1.

**Sheet 3 (Crapome analysis). A.** The two lists of proteins (HUGO gene name) are shown. In red, proteins discarded after the application of the Crapome filter (cut-off 25%, see main text for details) are shown. **B.** Proteins remaining after the crapome filter are shown. The Venn diagram shows the overlap among “unique” and “significant” proteins detected by co-IP (323 total proteins) and proximity-labeling of TBC1D22B (418 total proteins, as from Table S2, sheet 14) consisting in 53 common proteins. **C**. List of the 53 common proteins.

**Sheet 4 (GO terms**). The complete list of GO terms, obtained by Enrich (https://maayanlab.cloud/Enrichr/), enriched in the group of 323 proteins of the TBC1D22B interactome (221 significant, 102 unique), at an FDR < 0.05.

Data are in Supplementary Table 4.xlsx

**Table S5. Tertile analysis, supplementary data to Figure 3C**

The quantitation and statistical analysis of the data in Figure 3C are shown. At each time point, for each condition, the total number of cells analyzed, and the number of cells (*n*) falling in the upper tertile or the lower two tertiles are reported. Statistical analysis of the pairwise comparisons was performed with the Fisher’s test. Compared pairs are shaded differently to facilitate reading. Statistically significant differences are in red.

| **Time** | **Sample** | **Total no. of cells analyzed** | **Upper tertile** | | **Lower 2 tertiles** | | **P** |
| --- | --- | --- | --- | --- | --- | --- | --- |
|  |  |  | ***n* cells** | **%** | ***n* cells** | **%** |  |
| T0 | 22B | 72 | 0 | 0 | 72 | 100 | 1.0 |
|  | EV | 66 | 0 | 0 | 66 | 100 |  |
|  | RQ | 70 | 0 | 0 | 70 | 100 | 1.0 |
|  | EV | 66 | 0 | 0 | 66 | 100 |  |
|  | 22B | 72 | 0 | 0 | 72 | 100 | 1.0 |
|  | RQ | 70 | 0 | 0 | 70 | 100 |  |
| T20 | 22B | 78 | 18 | 23.1 | 60 | 76.9 | <.0001 |
|  | EV | 72 | 50 | 69.4 | 22 | 30.6 |  |
|  | RQ | 68 | 41 | 60.3 | 27 | 39.7 | 0.29 |
|  | EV | 72 | 50 | 69.4 | 22 | 30.6 |  |
|  | 22B | 78 | 18 | 23.1 | 60 | 76.9 | <.0001 |
|  | RQ | 68 | 41 | 60.3 | 27 | 39.7 |  |
| T120 | 22B | 68 | 3 | 4.4 | 65 | 95.6 | 0.62 |
|  | EV | 67 | 1 | 1.5 | 66 | 98.5 |  |
|  | RQ | 64 | 0 | 0 | 64 | 100 | 0.99 |
|  | EV | 67 | 1 | 1.5 | 66 | 98.5 |  |
|  | 22B | 68 | 3 | 4.4 | 65 | 95.6 | 0.25 |
|  | RQ | 64 | 0 | 0 | 64 | 100 |  |

**Table S6. Tertile analysis, supplementary data to Figure S3 CAL120 cells overexpressing EV, TBC1D22B and RQ**

The quantitation and statistical analysis of the data in Figure S3C are shown. At each time point, for each condition, the total number of cells analyzed, and the number of cells (*n*) falling in the upper tertile or the lower two tertiles are reported. Statistical analysis of the pairwise comparisons was performed with the Fisher’s test. Compared pairs are shaded differently to facilitate reading. Statistically significant differences are in red.

| **Time** | **Sample** | **Total no. of cells analyzed** | **Upper tertile** | | **Lower 2 tertiles** | | **P** |
| --- | --- | --- | --- | --- | --- | --- | --- |
|  |  |  | ***n* cells** | **%** | ***n* cells** | **%** |  |
| T0 | 22B | 38 | 0 | 0 | 38 | 100 | 1.0 |
|  | EV | 42 | 0 | 0 | 42 | 100 |  |
|  | RQ | 40 | 0 | 0 | 40 | 100 | 1.0 |
|  | EV | 42 | 0 | 0 | 42 | 100 |  |
|  | 22B | 38 | 0 | 0 | 38 | 100 | 1.0 |
|  | RQ | 40 | 0 | 0 | 40 | 100 |  |
| T30 | 22B | 39 | 3 | 7.7 | 36 | 92.3 | <.0001 |
|  | EV | 44 | 13 | 29.5 | 31 | 70.5 |  |
|  | RQ | 45 | 21 | 46.7 | 24 | 53.3 | 0.13 |
|  | EV | 44 | 13 | 29.5 | 31 | 70.5 |  |
|  | 22B | 39 | 3 | 7.7 | 36 | 92.3 | <.0001 |
|  | RQ | 45 | 21 | 46.7 | 24 | 53.3 |  |
| T120 | 22B | 35 | 0 | 0 | 35 | 100 | 1.0 |
|  | EV | 39 | 0 | 0 | 39 | 100 |  |
|  | RQ | 39 | 1 | 2.6 | 38 | 97.4 | 0.99 |
|  | EV | 39 | 0 | 0 | 39 | 100 |  |
|  | 22B | 35 | 0 | 0 | 35 | 100 | 0.99 |
|  | RQ | 39 | 1 | 2.6 | 38 | 97.4 |  |

**Table S7. Tertile analysis, supplementary data to Figure 4D BT549 cells silenced for TBC1D22B or TBC1D22A**

The quantitation and statistical analysis of the data in Figure 4D are shown. At each time point, for each condition, the total number of cells analyzed, and the number of cells (*n*) falling in the upper tertile or the lower two tertiles are reported. Statistical analysis of the pairwise comparisons was performed with the Fisher’s test. Statistically significant differences are in red.

| **Time** | **Sample** | **Total no. of cells analyzed** | **Upper tertile** | | **Lower 2 tertiles** | | **P** |
| --- | --- | --- | --- | --- | --- | --- | --- |
|  |  |  | ***n* cells** | **%** | ***n* cells** | **%** |  |
| T0 | siCTRL | 46 | 0 | 0 | 46 | 100 | 1.0 |
|  | si22B | 44 | 0 | 0 | 44 | 100 |  |
|  | siCTRL | 46 | 0 | 0 | 46 | 100 | 1.0 |
|  | si22A | 44 | 0 | 0 | 44 | 100 |  |
|  | si22A | 44 | 0 | 0 | 44 | 100 | 1.0 |
|  | si22B | 44 | 0 | 0 | 44 | 100 |  |
| T8 | siCTRL | 46 | 3 | 6.5 | 43 | 93.5 | <.0001 |
|  | si22B | 45 | 35 | 77.8 | 10 | 22.2 |  |
|  | siCTRL | 46 | 3 | 6.5 | 43 | 93.5 | 0.32 |
|  | si22A | 45 | 6 | 13.3 | 39 | 86.7 |  |
|  | si22A | 45 | 6 | 13.3 | 39 | 86.7 | <.0001 |
|  | si22B | 45 | 35 | 77.8 | 10 | 22.2 |  |
| T20 | siCTRL | 45 | 33 | 73.3 | 12 | 26.7 | 0.12 |
|  | si22B | 44 | 25 | 56.8 | 19 | 43.2 |  |
|  | siCTRL | 45 | 33 | 73.3 | 12 | 26.7 | 0.13 |
|  | si22A | 46 | 26 | 56.5 | 20 | 43.5 |  |
|  | si22A | 46 | 26 | 56.5 | 20 | 43.5 | 0.99 |
|  | si22B | 44 | 25 | 56.8 | 19 | 43.2 |  |
| T120 | siCTRL | 45 | 1 | 2.2 | 44 | 97.8 | 0.99 |
|  | si22B | 45 | 0 | 0 | 45 | 100 |  |
|  | siCTRL | 45 | 1 | 2.2 | 44 | 97.8 | 0.99 |
|  | si22A | 45 | 2 | 4.4 | 43 | 95.6 |  |
|  | si22A | 45 | 2 | 4.4 | 43 | 95.6 | 0.49 |
|  | si22B | 45 | 0 | 0 | 45 | 100 |  |

**Table S8. RAB selection from the TBC1D22B proximity interactome**

To select the candidate RABs for the silencing experiments shown in Figure 5, we focused on those present with high specificity and confidence in the 22B and RQ proximity-labeling datasets. We did not differentiate between proximity to 22B or RQ, since the effects of the mutation on the proximity or interaction of the GAP with the cognate RAB are unpredictable.

To achieve this, we elaborated a matrix scoring system, assigning each RAB +1 point for each occurrence in a 22B or RQ replicate with a peptide count ≥ 2, and -1 point for each occurrence in an EV or H_2_O_2_ control replicate. Top-ranking RABs (score > 5 points, boxed in red) included RAB8A, RAB1B, RAB5C, RAB35, RAB34 and RAB13. Additionally, we randomly selected some RABs with scores < 5 points (also with the finality of including some RABs present in the physical interactome list). Finally, we included RAB33A, despite its absence in our datasets, due to literature reports identifying TBC1D22B as a GAP for this RAB in yeast and in humans.^[2]^

The table lists all the RABs present in the proximity interactome along with their scores. The selected RABs are shaded in grey.

| **RAB** | **Arbitrary scores** | | | | | |
| --- | --- | --- | --- | --- | --- | --- |
|  | 22B | 22B/H2O2 | RQ | RQ/H2O2 | EV | Total Score |
| RAB8A | 4 | -1 | 4 | 0 | 0 | 7 |
| RAB1B | 4 | -1 | 4 | -1 | 0 | 6 |
| RAB5C | 4 | 0 | 4 | -2 | 0 | 6 |
| RAB35 | 3 | -1 | 4 | 0 | 0 | 6 |
| RAB34 | 3 | 0 | 3 | 0 | 0 | 6 |
| RAB13 | 4 | -1 | 4 | -2 | 0 | 5 |
| RAB6A | 4 | 0 | 4 | -3 | -2 | 3 |
| RAB11B | 4 | -2 | 4 | -1 | -2 | 3 |
| RAB6C | 0 | 0 | 4 | 0 | -1 | 3 |
| RAB18 | 3 | -1 | 4 | -2 | -1 | 3 |
| RAB32 | 3 | -1 | 4 | -1 | -2 | 3 |
| RAB9A | 0 | 0 | 3 | 0 | -1 | 2 |
| RAB10 | 4 | -3 | 4 | -2 | -1 | 2 |
| RAB27B | 0 | 0 | 2 | 0 | 0 | 2 |
| RAB1A | 4 | -3 | 4 | -2 | -2 | 1 |
| RAB2A | 4 | -2 | 4 | -3 | -2 | 1 |
| RAB7A | 4 | -2 | 4 | -3 | -2 | 1 |
| RAB14 | 4 | -2 | 4 | -3 | -2 | 1 |
| RAB21 | 3 | -3 | 0 | -1 | -1 | -2 |
| RAB33A | Not present in dataset | | | | | |

**Table S9. Tertile analysis, supplementary data to Figure 5C**

The quantitation and statistical analysis of the data in Figure 5C are shown. At each time point, for each condition, the total number of cells analyzed, and the number of cells (*n*) falling in the lower tertile or the upper two tertiles are reported. Statistical analysis of the pairwise comparisons was performed with the Fisher’s test. Statistically significant differences are in red.

|  |  |  | **Lower tertile** | | **Upper 2 tertiles** | |  |
| --- | --- | --- | --- | --- | --- | --- | --- |
| **Time** | **siRNA** | **Total no. of cells analyzed** | ***n* cells** | **%** | ***n* cells** | **%** | **P** |
| T20 | CTR | 69 | 5 | 7.3 | 64 | 92.7 | Ref. |
|  | RAB1B | 30 | 22 | 73.3 | 8 | 26.7 | <.0001 |
|  | RAB5C | 33 | 1 | 3.0 | 32 | 97.0 | 0.66 |
|  | RAB6A | 39 | 7 | 18.0 | 32 | 82.0 | 0.11 |
|  | RAB7A | 30 | 3 | 10.0 | 27 | 90.0 | 0.70 |
|  | RAB8A | 43 | 6 | 14.0 | 37 | 86.0 | 0.33 |
|  | RAB13 | 36 | 4 | 11.1 | 32 | 88.9 | 0.49 |
|  | RAB18 | 30 | 1 | 3.3 | 29 | 96.7 | 0.66 |
|  | RAB21 | 32 | 2 | 6.3 | 30 | 93.7 | 1.00 |
|  | RAB33A | 32 | 4 | 12.5 | 28 | 87.5 | 0.46 |
|  | RAB34 | 28 | 2 | 7.1 | 26 | 92.9 | 1.00 |
|  | RAB35 | 32 | 5 | 15.6 | 27 | 84.4 | 0.28 |

**Table S10. Transcriptional programs regulated by TBC1D22B and TBC1D22A in BC and BC cell lines**

**Sheet 1.** Genes up- and down-regulated in the comparison TBC1D22B-high and TBC1D22B-low (upper *vs*. lower quintile) in the METABRIC dataset.

**Sheet 2.** Genes up- and down-regulated in the comparison TBC1D22A-high and TBC1D22A-low (upper *vs*. lower quintile) in the METABRIC dataset.

**Sheet 3.** Genes up- and down-regulated in the comparison TBC1D22B/EV in BT549 transfectants.

**Sheet 4.** The sheet contains the complete list of the genes up-regulated or down-regulated in the METABRIC dataset (from sheet 1) and in the BT549 dataset (from sheet 3) and their overlap. The number of listed genes is lower than that reported in sheet 1 and 3 because only genes present in both datasets were considered to calculate the overlap.

**Sheet 5.** Enriched ontologies (FDR < 0.05) of the 159 down-regulated genes.

**Sheet 6.** Co-occurrence of down-regulation of the 159 genes in the METABRIC dataset. Co-occurrence was scored as significant at a q-value of < 0.05.

Data are in Supplementary Table 10.xlsx

**Table S11. siRNA oligos used in the study.** Gene IDs and catalogue numbers of siRNAs used in the study are listed.

| **Gene Symbol** | **GENE ID** | **Gene Accession** | **Catalogue ID** |
| --- | --- | --- | --- |
| CTRL |  |  | D-001810-10 |
| TBC1D22B | 55633 | NM_017772 | J-021109-09 |
|  |  |  | J-021109-10 |
|  |  |  | J-021109-11 |
|  |  |  | J-021109-12 |
| TBC1D22A | 25771 | NM_014346 | J-009319-09 |
|  |  |  | J-009319-10 |
|  |  |  | J-009319-11 |
|  |  |  | J-009319-12 |
| RAB1B | 81876 | NM_030981 | J-008958-09 |
|  |  |  | J-008958-10 |
|  |  |  | J-008958-11 |
|  |  |  | J-008958-12 |
| RAB5C | 5878 | NM_001252039 | J-004011-07 |
|  |  |  | J-004011-08 |
|  |  |  | J-004011-09 |
|  |  |  | J-004011-10 |
| RAB6A | 5870 | NM_001243718 | J-008975-07 |
|  |  |  | J-008975-08 |
|  |  |  | J-008975-09 |
|  |  |  | J-008975-10 |
| RAB7A | 7879 | NM_004637 | J-010388-05 |
|  |  |  | J-010388-06 |
|  |  |  | J-010388-07 |
|  |  |  | J-010388-08 |
| RAB8A | 4218 | NM_005370 | J-003905-05 |
|  |  |  | J-003905-06 |
|  |  |  | J-003905-07 |
|  |  |  | J-003905-08 |
| RAB13 | 5872 | NM_001272038 | J-008389-05 |
|  |  |  | J-008389-06 |
|  |  |  | J-008389-07 |
|  |  |  | J-008389-08 |
| RAB18 | 22931 | NM_001256410 | J-010824-05 |
|  |  |  | J-010824-06 |
|  |  |  | J-010824-07 |
|  |  |  | J-010824-08 |
| RAB21 | 23011 | NM_014999 | J-009450-05 |
|  |  |  | J-009450-06 |
|  |  |  | J-009450-07 |
|  |  |  | J-009450-08 |
| RAB33 | 9363 | NM_004794 | J-008206-05 |
|  |  |  | J-008206-06 |
|  |  |  | J-008206-07 |
|  |  |  | J-008206-08 |
| RAB34 | 83871 | NM_001142624 | J-009735-05 |
|  |  |  | J-009735-06 |
|  |  |  | J-009735-07 |
|  |  |  | J-009735-08 |
| RAB35 | 11021 | NM_001167606 | J-009781-05 |
|  |  |  | J-009781-06 |
|  |  |  | J-009781-07 |
|  |  |  | J-009781-08 |

**Table S12. RNAseq data of BT549 cells overexpressing TBC1D22B or the EV control; data used to prepare Fig. 8B-D.** The entire dataset, 38,979 genes, is reported for RNAseq experiments perfomed in triplicate (#1, #2, #3) in the indicated conditions. The pipeline of analysis is described in details in the Experimental section.

**Sheet 1:** RAW data.

**Sheet 2:** TMMs (Trimmed Mean of M-values).

**Sheet 3:** TPMs (Transcripts Per Kilobase Million).

Data are in Supplementary Table 12.xlsx


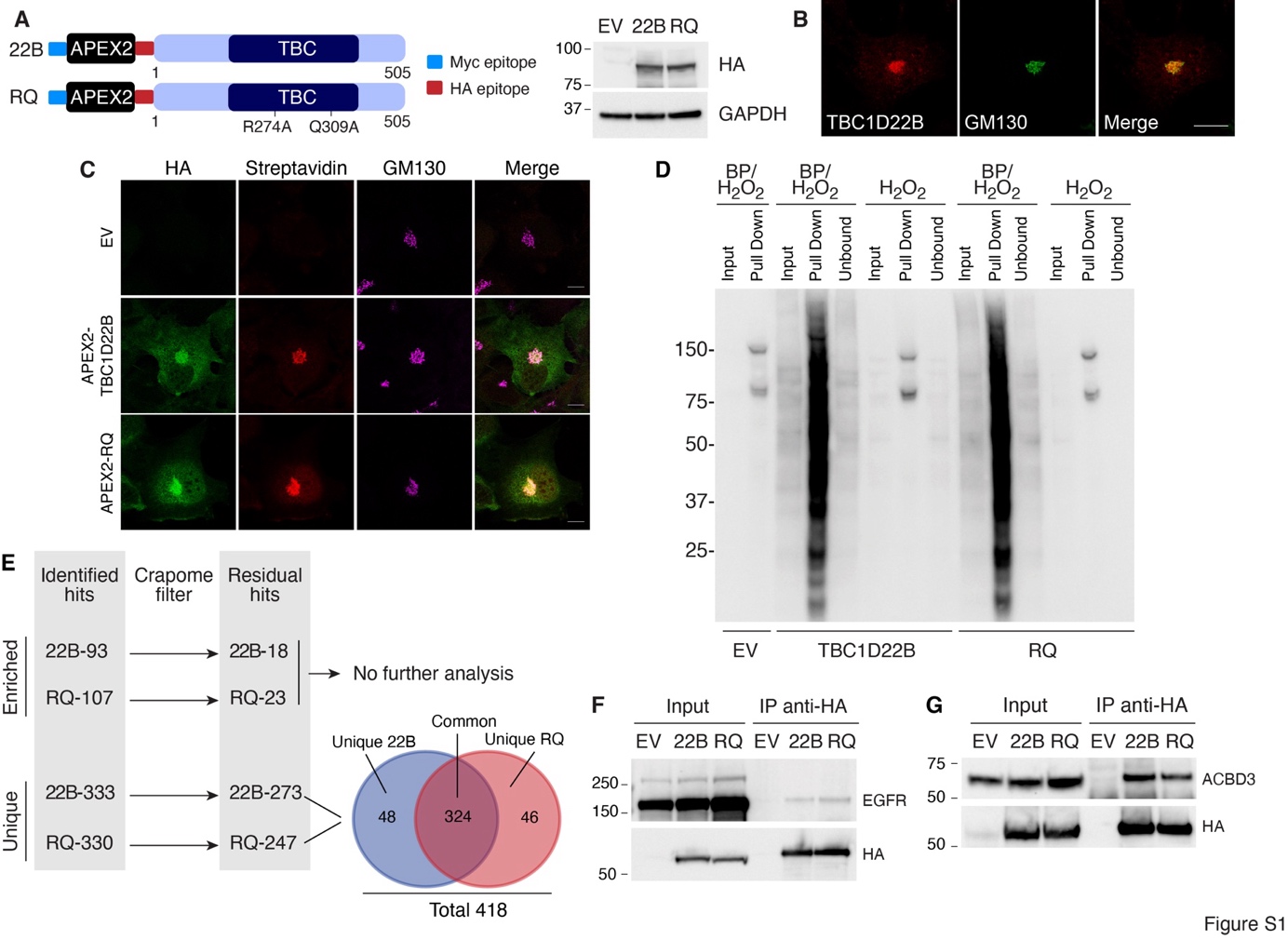


**Figure S1**. **proximity biotinylation with APEX2-TBC1D22B and APEX2-TBC1D22B-RQ. A.** Left, schematic diagram of the expression vectors for APEX2-TBC1D22B (22B) and APEX2-TBC1D22B-RQ (RQ), with amino acid positions of the TBC1D22B sequence and mutations (RQ) indicated. Right, immunoblots (IB) showing expression levels (anti-HA) of APEX2-TBC1D22B and APEX2-TBC1D22B-RQ in stably transduced BT549 cells. EV, empty vector control. GAPDH, loading control. **B.** Confocal images showing the co-localization of endogenous TBC1D22B (in red) with the Golgi marker GM130 (in green), resulting in yellow staining in the merged images (merge). Bar, 10 μm. **C.** Biotinylating activity of APEX2-TBC1D22B fusion proteins in BT549 cells. Cells stably expressing APEX2-TBC1D22B, APEX2-TBC1D22B-RQ (RQ), or EV (empty vector), were subjected to biotinylation and stained as indicated. Streptavidin-555 (in red) was used to visualize biotinylated proteins. Anti-HA was used to evaluate APEX2-TBC1D22B fusion protein expression levels. Anti-GM130 (in magenta) revealed the Golgi apparatus. Bar, 10 μm. **D.** Streptavidin pulldown of biotin-labeled proteins from BT549 cells expressing the indicated constructs (EV, TBC1D22B, RQ; see Experimental section for details on sample preparation). Treatments are shown on top (BP, biotin-phenol, H_2_O_2_ hydrogen peroxide). For each condition, the input (5 μg of total lysates), streptavidin pull-down (1/10 of the total), and unbound samples (same volume as input) are shown. IB anti-Streptavidin-HRP. **E.** The procedure used to select the hits for pathway analysis is shown. For the 22B proximity proteome, “enriched” proteins had to be enriched in both the 22B *vs*. EV and the 22B *vs*. H_2_O_2_-only comparisons with a p-adjusted value of ≤ 0.05. In addition, they had to pass a peptide filter of ≥ 2 peptides in all 22B replicates (Table S2, sheets 1-3). “Unique” proteins were detected in all the 22B experimental replicas (4 of 4) and absent in all controls (4 replicas of both EV and H_2_O_2_-only), and had to pass the ≥ 2 peptide filter in all 22B replicates (Table S2, sheets 4-6). The same criteria were used to identify “enriched” and “unique” proteins in the RQ proximity proteome (Table S2, sheets 7-9 and 10-12, respectively). For the “Crapome filter”, a cut-off of 25% was used, thereby excluding proteins present in > 25% of instances in the crapome database. To generate the final proteome, we relaxed the stringency criteria including proteins that appear in 1 replicate of 1 control obtaining a total number of 418 hits in 22B and RQ. The overlap between 22B and RQ is shown in the Venn diagram on the right. **F, G**. Total lysates from BT549 cells, stably expressing HA-tagged TBC1D22B (22B) or the TBC1D22B-RQ mutant (RQ), were immunoprecipitated (IP) with anti-HA and IB as shown on the right. Control cells were transfected with empty vector (EV). In this and all subsequent figures, molecular weight markers (on the left) are shown in KDa.

**
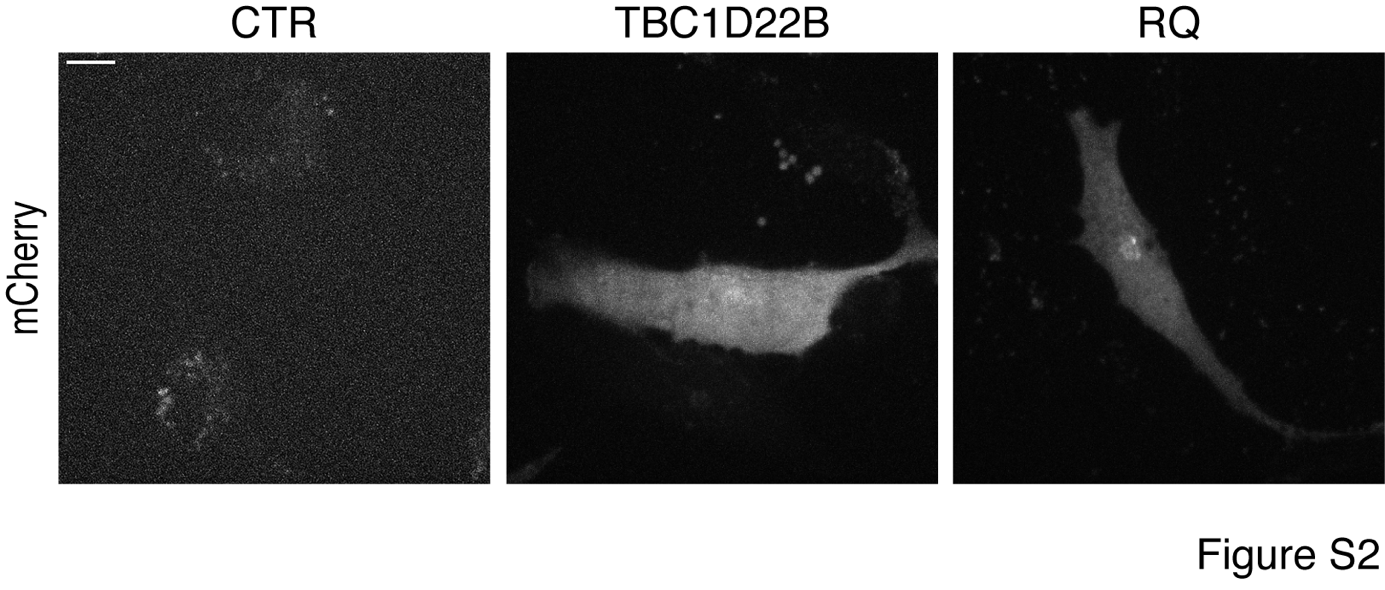
**

**Figure S2. Supplementary data to Movies 1, 2, and 3.** Snapshot images of mCherry fluorescence taken from the Movies before addition of biotin showing the expression of mCherry-TBC1D22B or mCherry-RQ in BT549 cells co-transfected with the RUSH construct (Str-KDEL_SBP-EGFP-GPI, the GFP-GPI fluorescence is recorded in the Movies). Control cells (CTR) were transfected with the RUSH construct Str-KDEL_SBP-EGFP-GPI alone. Bar, 10 μm.


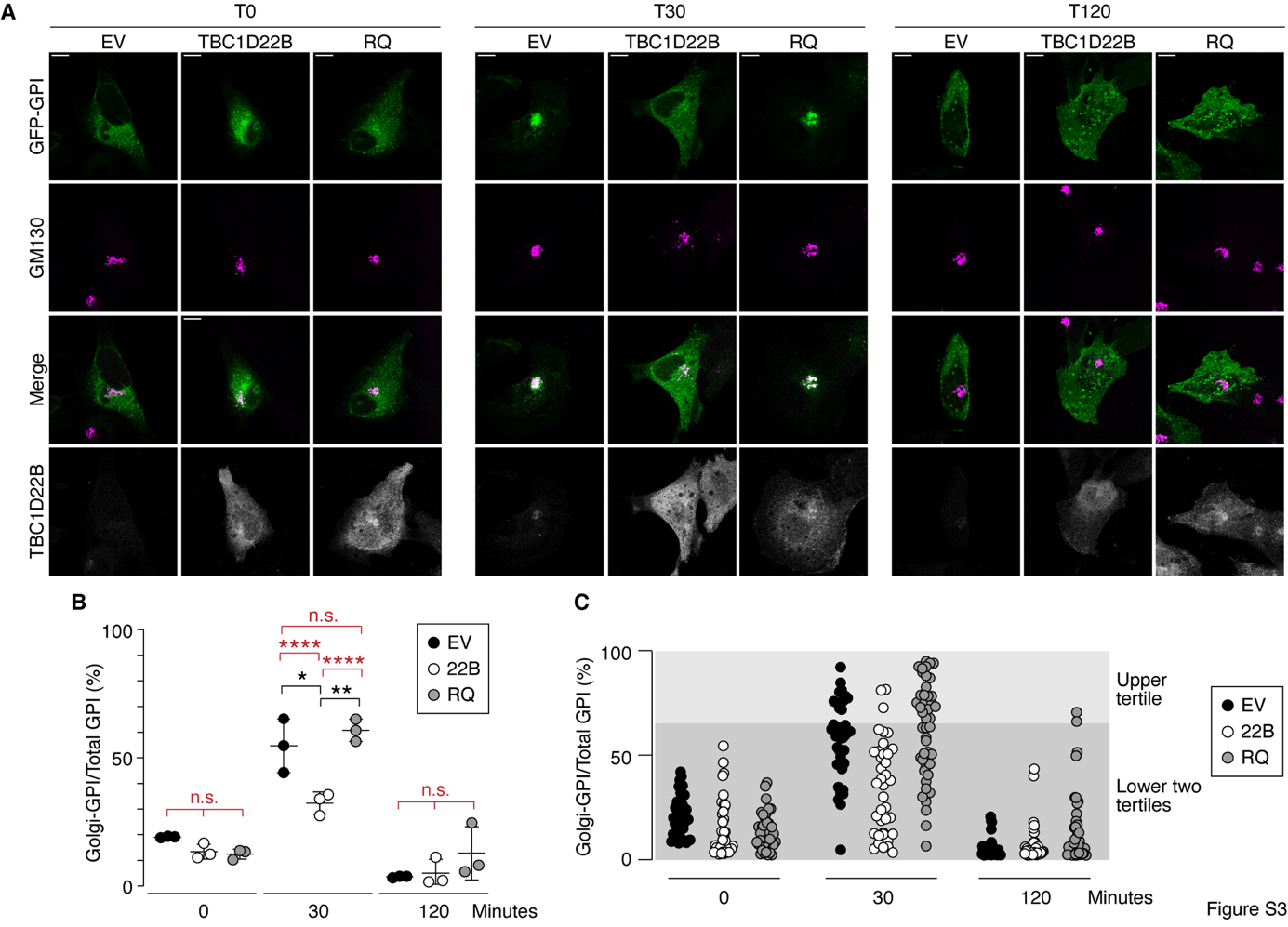


**Figure S3. Effect of TBC1D22B overexpression on ER-to-Golgi transport in CAL120 cells. A.** RUSH assays in CAL120 cells stably expressing HA-TBC1D22B or HA-TBC1D22B-RQ (RQ) or empty vector (EV) control. Cells were transfected with the Str-KDEL_SBP-EGFP-GPI RUSH construct, then treated with biotin to release the GFP-GPI reporter. Time points after biotin addition are indicated on top. In setting up experiments done using the RUSH system in the CAL120 cell line (not shown) relocalization of the GFP-GPI reporter to the Golgi occurred with a slightly slower kinetic compared to BT549 cells, reaching the peak 30 min after the addition of biotin in not transduced, control, cells. Therefore, time course of release used for the experiments done in CAL120 cells was T0, T30 and T120 min. Images show GFP-GPI epifluorescence (green), and IF staining for the Golgi marker GM130 (magenta) and for TBC1D22B (grey). Merge results from the overlap of the GFP-GPI epifluorescence and the GM130 staining. Bar, 10 μm **B.** Quantitation of the experiment shown in A. The percentage of Golgi-localized GFP-GPI, normalized to the total GFP-GPI signal per cell, is plotted at time 0, 30 and 120 min after biotin addition. Data represent the mean ± SD of four independent experiments, in which ~8-15 cells/experiment/condition were analyzed. Significance (black asterisks) was calculated with the t-test. Red asterisks refer to the statistical analysis performed on the tertile distribution shown in panel C. *, p < 0.05, **, p < 0.01, **** p < 0.0001, n.s., not significant. **C.** Stratification of the entire dataset of % Golgi-GPI/Total-GPI per cell is shown (~40 cells/conditions). The upper tertile shows cells with a Golgi-GPI > 66%; the lower two tertiles show cells with a Golgi-GPI ≤ 66%. Statistical analysis was performed with the Fisher’s test and is reported in Table S6 (also as red asterisks in panel B).


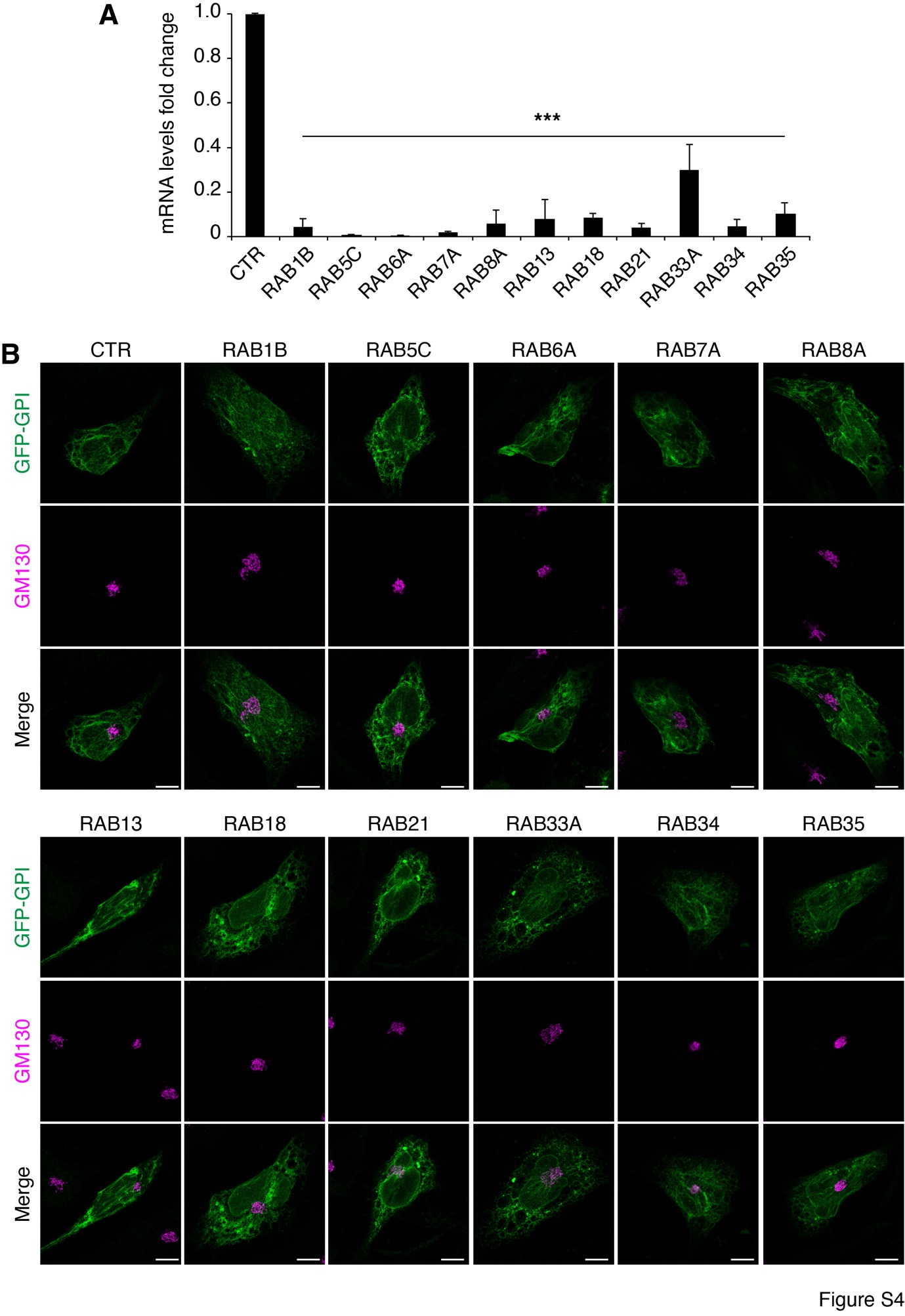


**Figure S4. Supplementary data to Figure 5 of the main text. A.** Total cellular RNA was extracted from BT549 cells silenced with control oligos (CTR) or pools of 4 oligos targeting each of the indicated RABs. Silencing efficiency was verified at the mRNA levels by qPCR. Bar graph shows the mean fold-change + SD of two independent experiments performed in technical triplicates after normalization to CTR. Statistical analysis was performed with the t-test, ***, p < 0.001. **B.** RUSH assays in BT549 cells silenced for the indicated RABs. Images correspond to T0 (T20 is shown in Figure 5A). Images here and in Figure 5A were taken from the same experiment with identical settings. GFP-GPI epifluorescence (green) and IF staining of the Golgi marker GM130 (magenta) are shown. Bar, 10 μm.

**SUPPLEMENTARY LEGENDS TO MOVIES**

**Movie 1. *In vivo* RUSH assay in BT549 CTR cells.** BT549 cells transfected with the Str-KDEL_SBP-EGFP-GPI construct. Time 0 corresponds to the addition of biotin to release the reporter. The signal corresponds to epifluorescence of the GFP-GPI reporter. Time is in min.

**Movie 2. *In vivo* RUSH assay in BT549 mCherry-TBC1D22B cells.** BT549 cells co-transfected with the Str-KDEL_SBP-EGFP-GPI and mCherry-TBC1D22B constructs. Expression of mCherry-TBC1D22B is shown in Figure S2. Time 0 corresponds to the addition of biotin to release the reporter. The signal corresponds to epifluorescence of the GFP-GPI reporter. Time is in min.

**Movie 3. *In vivo* RUSH assay in BT549 mCherry-RQ cells.** BT549 cells co-transfected with the Str-KDEL_SBP-EGFP-GPI and mCherry-RQ constructs. Expression of mCherry-RQ is shown in Figure S2. Time 0 corresponds to the addition of biotin to release the reporter. The signal corresponds to epifluorescence of the GFP-GPI reporter. Time is in min.

**REFERENCES**

# 1. J. Cox, N. Neuhauser, A. Michalski, R. A. Scheltema, J. V. Olsen, M. Mann, "Andromeda: A Peptide Search Engine Integrated into the MaxQuant Environment", *J Proteome Res* (2011): 10 (4), 1794, <https://doi.org/10.1021/pr101065j>.

# 2. a) S. Albert, E. Will, D. Gallwitz, "Identification of the catalytic domains and their functionally critical arginine residues of two yeast GTPase‐activating proteins specific for Ypt/Rab transport GTPases", *EMBO J* 1999, *18* (19), 5216, <https://doi.org/10.1093/emboj/18.19.5216>; b) X. Pan, S. Eathiraj, M. Munson, D. G. Lambright, "TBC-domain GAPs for Rab GTPases accelerate GTP hydrolysis by a dual-finger mechanism", *Nature* (2006): 442 (7100), 303, <https://doi.org/10.1038/nature04847>.
